# Isotemporal substitution from sedentary behavior or sleep to physical activity: associations with depression risk in older adults—a systematic review and meta-analysis

**DOI:** 10.3389/fpsyg.2025.1682987

**Published:** 2026-01-14

**Authors:** Jiali Wang, Yilin Wang, Yue Wang, Yuheng Zhang, Junjie Liu, Songtao Lu

**Affiliations:** 1 School of Physical Education, Wuhan University of Science and Technology, Wuhan, China; 2 Faculty of Artificial Intelligence in Education, Central China Normal University, Wuhan, China; 3 China Football College, Beijing Sport University, Beijing, China

**Keywords:** depression, isotemporal substitution model, meta-analysis, older adults, physical activity, sedentary behavior, sleep

## Abstract

**Background and aims:**

As global population aging intensifies, mental health issues in older adults are increasingly prominent, with depression being particularly prevalent and detrimental. The study investigated how substituting sedentary behavior (SB) and sleep (SLP) with physical activity (PA) affects depression risk in this population.

**Methods:**

Meta-analysis was conducted by searching four databases: PubMed, Scopus, SPORTdiscus, and PsycINFO (via EBSCOhost platform) for relevant studies published until January 2025. Regression coefficients (β) with 95% confidence intervals (CIs) for depressive symptoms were estimated. Publication bias was assessed using funnel plots and Egger’s tests, and heterogeneity was evaluated using Q tests and the I^2^ statistic. All statistical analyses were performed using STATA software (version 18.0).

**Results:**

Among 18,912 participants (53.45% female, ≥60 years) across nine studies, replacing SB with MVPA significantly reduced depression (β = −0.12, 95% CI: −0.20, −0.04), subgroup analyses indicated that reallocating 10, 30 and 60 min/day of SB to MVPA (*β* = −0.06, 95% CI = −0.19, 0.08; *β* = −0.14, 95% CI = −0.25, −0.02; *β* = −0.34, 95% CI = −0.59, −0.09), while light PA (LPA) showed no significant effect (*β* = −0.02, 95% CI = −0.08, 0.03). Replacing SB with SLP marginally reduced depression (*β* = −0.06, 95% CI = −0.10, 0.00). Substituting SLP with MVPA also lowered risk (*β* = −0.16, 95% CI: −0.31, −0.01).

**Conclusion:**

Substituting SB and SLP with MVPA is significantly associated with a reduction in depression, whereas no significant association is observed when replaced by LPA.

**Systematic review registration:**

https://www.crd.york.ac.uk/PROSPERO/display_record.php?RecordID=546666, identifier CRD42024546666.

## 1 Introduction

As the global trend of population aging increases, mental health problems are becoming more prominent among older adults, with depression and anxiety being particularly common. According to the World Health Organization (WHO), approximately 3.8% of the global population suffers from depression, and the prevalence is as high as 5.7% amongst older adults aged 60 years and over (WHO, 2023). Even more concerningly, it is predicted that by 2030, depression will be the leading cause of disease burden globally, with lasting and heavy burdens across the lifespan of the sufferer, as well as on the family (WHO, 2011). However, depression in older adults is often overlooked, and its treatment rates are relatively low. This situation not only leads to impaired functioning in basic activities of daily living but also significantly reduces the quality of life of older adults. Therefore, focusing on and developing effective intervention and counseling strategies for depression in older adults is extremely important for alleviating depressive symptoms and enhancing the quality of life and well-being.

PA is an important modifiable lifestyle factor that improves mental health. A large number of studies have confirmed that PA is beneficial for alleviating depressive symptoms in older adults (Birch et al., 2016; Hemmeter and Ngamsri, 2022; Ku et al., 2012; Lampinen et al., 2000; Lee et al., 2014; Pasco et al., 2011; Paulo et al., 2016; Strawbridge et al., 2002; Sun and Biddle, 2002; Wang and Zheng, 2019; Yoshida et al., 2015; Zhang et al., 2021). Based on these findings, some researchers have further explored the positive effects of physical exercise on alleviating depressive symptoms in this population (Bridle et al., 2012; Ku et al., 2017; Sjosten and Kivela, 2006; Yang et al., 2023). Additionally, studies have compared the alleviation effects of different exercise models on depressive symptoms in older adults (Liang et al., 2021; Joshi et al., 2016; Miller et al., 2020). According to the Global recommendations on PA for health, all adults should do at least 150 to 300 min a week of moderate-intensity, or 75 to 150 min a week of vigorous-intensity aerobic PA, or an equivalent combination of moderate-intensity and vigorous-intensity aerobic PA (WHO, 2010). Research on the effects of different intensities of PA on depression in older adults has primarily focused on walking (Hernandez et al., 2013; Smith et al., 2010), leisure-time PA (Chen et al., 2012; Chi et al., 2016; Hamer et al., 2009), LPA (Felez-Nobrega et al., 2021; Ku et al., 2018), MVPA (Laird et al., 2023; McDowell et al., 2018).

However, SB is another critical factor influencing mental health. Existing research suggests that SB is positively associated with the risk of depression in older adults (Del Pozo Cruz et al., 2020; Eriksson et al., 2020; Tsutsumimoto et al., 2017), which aligns with the WHO recommendations on SB (WHO, 2020). Notably, there are differences in specific categorizations of SB. For example, passive SB, such as watching TV, significantly increase the risk of depression (Hamer et al., 2013; Hamer and Stamatakis, 2014; Li et al., 2024), whereas active or positive SB, such as surfing the internet or reading, may reduce the risk of depression (Hamer and Stamatakis, 2014; Jiang et al., 2024; Li et al., 2024).

As research continues, a few researchers have begun to explore the use of a combination of PA and SB accordingly to improve and prevent depressive symptoms in older adults. Studies have shown that reducing SB and increasing PA can reduce the risk of depression in older adults (Abe et al., 2019; Andrade-Gómez et al., 2018; Santos et al., 2017). In isotemporal substitution models, replacing SB with LPA or MVPA is usually associated with a reduced risk of depressive symptoms in older adults (Chen et al., 2024; Wei et al., 2019; Yasunaga et al., 2018). But, a study has found that replacing SB with LPA or MVPA is associated with improvements in anxiety symptoms, while the effects on depressive symptoms are inconsistent (Tully et al., 2020).

Moreover, the durations of PA, SB, and SLP are interrelated, as these durations sum up to a total of 24 h in a day. Therefore, an increase in any behavior will necessarily lead to a decrease in time spent on one or more other behaviors (Pedišić, 2014). Despite this interrelationship, fewer studies have used isotemporal substitution models to analyze the combined effects of PA, SB, and SLP on depression. Some studies have begun to focus on the relationship between SLP, SB, PA, and mental health in older adults (Hofman et al., 2022; Kandola et al., 2021; Meneguci et al., 2024). Compositional isotemporal analyses showed that hypothetically replacing SLP, SB, or LPA with MVPA could result in modest but significant improvements on mental health indicators (Cabanas-Sánchez et al., 2021). Therefore, a systematic review and meta-analysis are warranted to synthesize the extant research findings employing the isotemporal substitution methodology. Exploring the redistribution of time for PA, SB, and SLP would be beneficial in improving depression in older adults and reflects the relevance of this study.

## 2 Methods

The systematic review and meta-analysis undertaken in this investigation were meticulously executed in compliance with the Preferred Reporting Items for Systematic Reviews and Meta-Analyses (PRISMA) guidelines (Page et al., 2021). The PRISMA 2020 checklist is provided as Supplementary File 2. Isotemporal Substitution was the Gold Standard Model for Physical Activity Epidemiology (Mekary and Ding, 2019), so this model was adopted. The study protocol for this systematic review was registered in the PROSPERO International Prospective Register of Systematic Reviews with the registration number CRD42024546666.

### 2.1 Search strategy

A search was conducted through the PubMed, PsycINFO, Scopus, and SPORTdiscus electronic databases. The last search was conducted in January 15, 2025. No restrictions were placed on the publication date. The following search terms and keywords were used: (Joint or Isotemporal substitution or Substitutions or Time-use or Replace or Reallocation) and ([physical activity or physical activities or sport or exercise or aerobic exercise] or [sedentary lifestyle or sedentary behavior or sedentary time] or [sleep or sleep behavior or sleep time]) and (depression or depressive symptoms or mood depression or depressive mood). In addition, the catalog of references was examined to detect studies potentially eligible for inclusion. The complete search strategy, including all terms and syntax used for each database, is provided in Supplementary Tables S1–S4.

### 2.2 Eligibility criteria

Studies that met the following eligibility criteria were included in the systematic review and meta-analysis:(1) cohort, cross-sectional, and case–control studies; (2) reported objective (e.g., accelerometer) or self-reported (e.g., IPAQ, GPAQ, PSQI, et al.) measures of PA, SLP, and SB. Studies utilizing either type of measurement were eligible, and the measurement method was considered during data synthesis; (3) Studies were required to have operationalized the isotemporal substitution model. To meet this criterion, the analysis must have been based on a regression model that included time-use variables for multiple behavioral domains (e.g., SB, LPA, MVPA, SLP) while excluding one as the reference category. The primary outcome of the model must have been a quantitative estimate (e.g., beta coefficient) of the theoretical effect of isotemporally replacing a specified unit of sedentary time (e.g., 10 min/day, 30 min/day, 60 min/day) with an equivalent duration of LPA, MVPA, or SLP. Analyses that evaluated the effect of a single activity without modeling time displacement were excluded; (4) focused on older adults with age of 60 years or older without being specific disease group; and (5) reported primary research findings. Literature reviews, editorial comments, conference papers, book reviews, book chapters, postgraduate theses, and doctoral dissertations were excluded from the inclusion criteria. If a study had multiple publications, the most recent and complete publication data were included.

### 2.3 Search outcomes

All of the references from the search were downloaded into a bibliographic software package (EndNote X9), and duplicates were removed. The study selection process was independently conducted by two reviewers (Y. Wang. and Y. Zhang.). The process consisted of two stages: first, titles and abstracts were screened against the eligibility criteria to exclude clearly irrelevant records; second, the full texts of all potentially relevant publications—including those where eligibility remained uncertain—were thoroughly assessed against the predefined inclusion and exclusion criteria. Any disagreements between the two reviewers at either stage were resolved through discussion, and when consensus could not be reached, a third senior reviewer (S. Lu.) was consulted to make a final decision.

### 2.4 Quality assessment

Each study’s quality was assessed using an adjusted format of the Newcastle Ottawa quality assessment scale (Wells et al., 2000). This scale contains eight items categorized into three domains (selection, comparability, and exposure). A star system enables semiquantitative assessment of study quality, such that the highest-quality studies are awarded a maximum of one star per item except for the comparability domain, which allows allocating two stars. Thus, the score ranges from zero to ten stars (the maximum score for cohort and cross-sectional studies was nine and ten, respectively). If appropriate methods were reported, the criterion received a single star. A higher score suggests a lower risk of bias. Two reviewers independently assessed all included studies. Differences in opinion and discrepancies were resolved through discussion with a third author.

### 2.5 Data extraction

The following data were extracted independently by two authors using a standardized data extraction form. The meta-analysis was restricted to studies that explicitly applied the isotemporal substitution model. When studies reported multiple model results, only isotemporal substitution estimates were extracted. The form captured the following information: first author, publication year, country of origin, study population sample characteristics, participant demographics (including age [reported as mean with standard deviation], gender, and socioeconomic status, etc.), study type, age group classifications, beta coefficients (β) with 95% confidence intervals, substitution type and duration, measures of SLP, SB, and PA, depression outcomes (specifying the assessment instrument used, such as CES-D, GDS, or PHQ-9, etc.), and effect measures (see Supplementary Table S5). Any discrepancies in data extraction were resolved through discussion and consensus among at least three authors.

### 2.6 Synthesis

All analyses were performed using STATA software (version 18.0), and all tests were two-sided. We performed a two-step meta-analysis of categorical and continuous variables to assess the association between PA, SB, SLP, and the risk of depression. Pooled results are expressed as β with 95% Cis (see Supplementary Table S6). Pooled analyses were categorized into cohort and cross-sectional studies based on the study type, and pooled ORs were estimated using a random-effects model. Heterogeneity was assessed using the I^2^ statistic as the study’s variation percentage, with I^2^ values of 25–49%, 50–74%, and ≥75% indicating low, medium, and high heterogeneity, respectively (Higgins et al., 2019). The *p*-value for assessing heterogeneity was set at 0.05. Egger and Begg’s tests were used to determine whether publication bias existed (Higgins and Thompson, 2002). For the sensitivity analysis, each study was removed individually to check whether the combined effect of the remaining studies had changed. Subgroup meta-analysis was conducted by study type, sex, age, study area, study quality, and adjustment for confounding factors. Meta-regression was used to examine heterogeneity among studies.

We performed random-effects meta-analyses using the restricted maximum likelihood estimator. Between-study heterogeneity was quantified using I^2^ and τ^2^ statistics. To assess the robustness of findings and address methodological concerns raised by reviewers, we conducted several sensitivity analyses: (1) calculated 95% prediction intervals to estimate the range of true effects in future studies; (2) performed leave-one-out analyses to identify studies with disproportionate influence; (3) computed Cook’s distance statistics to formally assess study influence; (4) conducted trim-and-fill analysis as a sensitivity test for publication bias; and (5) considered p-curve analysis to evaluate evidentiary strength. We explicitly discuss multiplicity issues arising from multiple substitution contrasts within studies.

## 3 Results

### 3.1 Study selection

**Figure 1:**
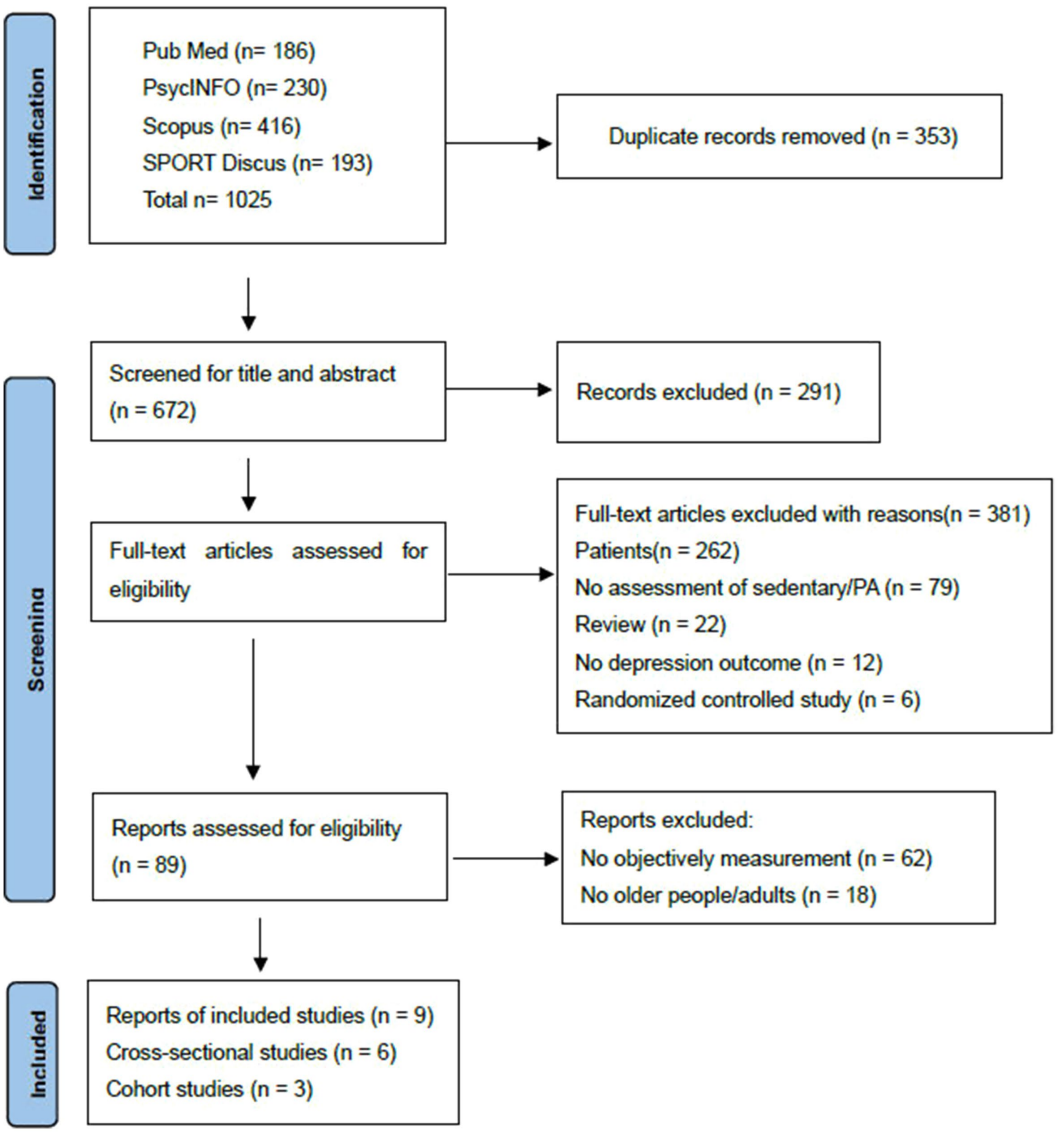
PRISMA diagram.

The initial search identified 1,025 articles. After removing 353 duplicates, 672 articles underwent title and abstract screening, resulting in the exclusion of 291 irrelevant publications. The remaining 89 articles were subjected to full-text review, of which 80 were excluded for not employing an isotemporal substitution model or lacking relevant outcome measures. Consequently, nine studies were ultimately included in this systematic review and meta-analysis. The detailed study selection process and reasons for exclusion are presented in Figure 1.

### 3.2 Study characteristics

Nine studies included a total of 18,912 participants. The sample size ranged from 139 individuals to 8,374 individuals. Sex was evenly distributed (women, 53.45%), and the mean age was 67.16 years. Three of the studies were cohort studies and observational investigations, and six were cross-sectional studies. The characteristics of these studies are summarized in Supplementary Table S7. The methodological quality of the included studies is presented in Supplementary Table S8. In total, 8 of the 9 studies were of high quality. All the included studies were rated with a score of ≥ 7 stars, indicating that they are of high quality and have a low risk of bias.

### 3.3 Isotemporal substitution of SB to LPA and depression risk

**Figure 2:**
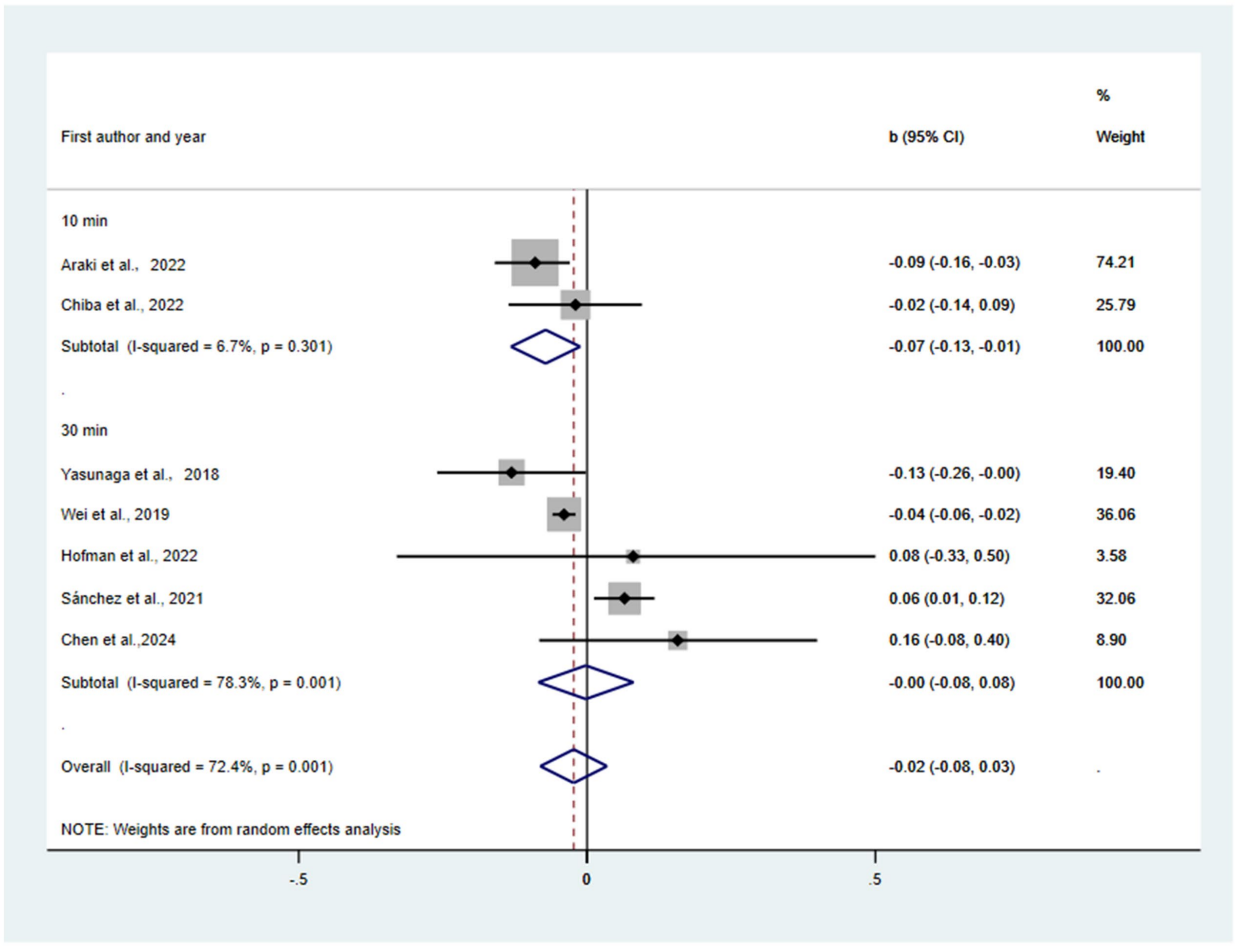
Pooled effect size of substitution of SB to LPA on the risk of depression.

Substitution of 10, 30 min/day, all sedentary time with LPA was predicted to be associated with reductions in depression (*β* = −0.07, 95% CI = −0.13, −0.01, *p* = 0.019; *β* = −0.00, 95% CI = −0.08, 0.08, *p* = 0.959; β = −0.02, 95% CI = −0.08, 0.03, *p* = 0.433) (see Figure 2). There was not a statistically significant publication bias in the funnel plot (see Supplementary Figure 1) and Egger’s test (*p* = 0.49). Substantial heterogeneity was observed (I^2^ = 72.4%, τ^2^ = 0.0018). The 95% prediction interval ranged from −0.15 to 0.11, indicating that the true effect in similar future studies could be beneficial, null, or even harmful. Sensitivity analyses supported the robustness of this null finding: leave-one-out analyses (see Supplementary Figure 5) showed no single study disproportionately influenced the pooled estimate (largest change: b = −0.04 after excluding Hofman et al., 2022), and Cook’s distance statistics (see Supplementary Table S9) confirmed no overly influential studies (all D < 0.571 threshold). Trim-and-fill analysis (see Supplementary Figure 9) estimated one missing study and yielded a similar non-significant effect (b = −0.03, 95% CI: −0.09 to 0.03). Consideration of multiplicity due to multiple substitution contrasts within the isotemporal framework and p-curve analysis (see Supplementary Figure 13) suggesting limited evidentiary strength further support a cautious interpretation of these null results.

### 3.4 Isotemporal substitution of SB to MVPA and depression risk

**Figure 3:**
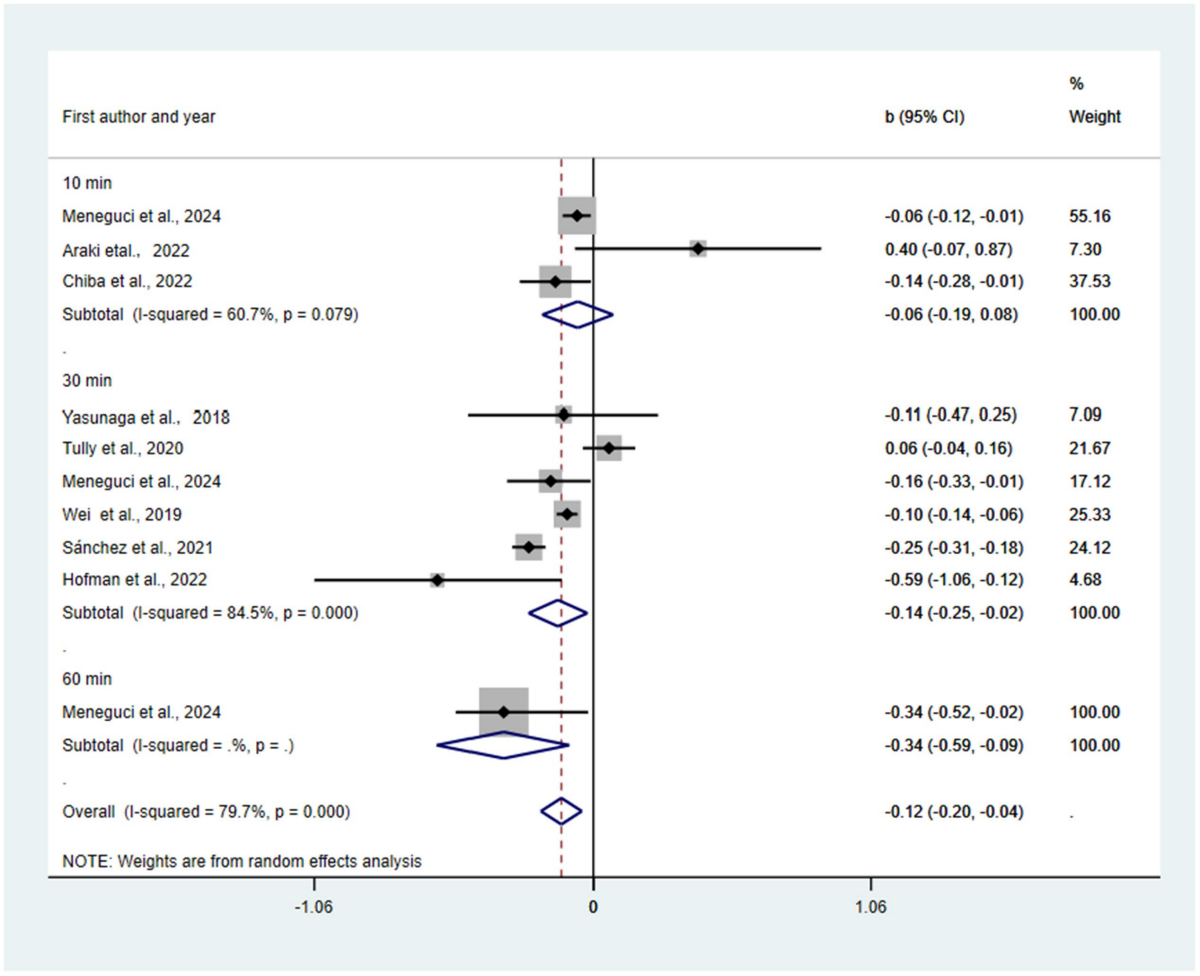
Pooled effect size of substitution of SB to MVPA on the risk of depression.

Substitution of 10, 30, and 60 min/day, all sedentary time with MVPA was predicted to be associated with reductions in depression (*β* = −0.06, 95% CI = −0.19, 0.08, *p* = 0.39; *β* = −0.14, 95% CI = −0.25, −0.02, *p* = 0.018; *β* = −0.34, 95% CI = −0.59, −0.09; *β* = −0.12, 95% CI = −0.20, −0.04, *p* = 0.002) (see Figure 3). There was not a statistically significant publication bias in the funnel plot (see Supplementary Figure 2) and Egger’s test (*p* = 0.71). The pooled effect of replacing SB with MVPA was −0.12 (95% CI: −0.20 to −0.04; I^2^ = 79.7%, τ^2^ = 0.0123). The 95% prediction interval ranged from −0.40 to 0.16, indicating considerable uncertainty about the effect magnitude and direction in future studies. Leave-one-out analyses (see Supplementary Figure 6) demonstrated robustness, with all recalculated estimates remaining statistically significant (range: −0.16 to −0.10). Cook’s distance diagnostics (see Supplementary Table S10) identified no influential studies (all values < 0.4). Trim-and-fill analysis (see Supplementary Figure 10) suggested potential publication bias, with the R0 estimator imputing three missing studies on the left side of the funnel plot (smaller studies with stronger protective effects). Among the six statistically significant studies, p-curve analysis (see Supplementary Figure 14) indicated strong evidential value (right-skewness test: *p* < 0.001) with minimal evidence of p-hacking (flatness test: *p* = 0.15).

### 3.5 Isotemporal substitution of SB to SLP and depression risk

**Figure 4:**
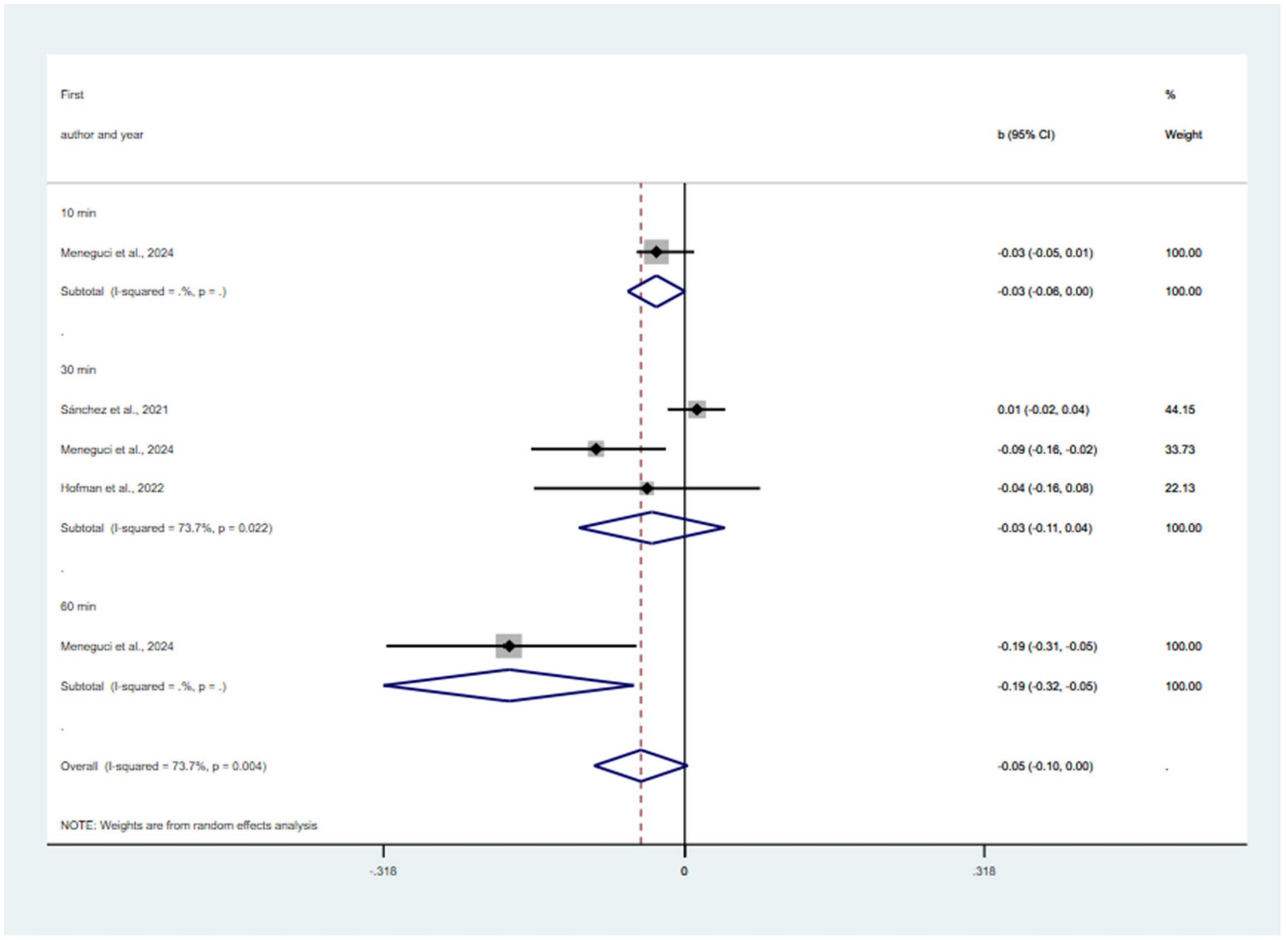
Pooled effect size of substitution of SB to SLP on the risk of depression.

Substitution of 10, 30, 60 min/day, all sedentary time with SLP was predicted to be associated with reductions in depression (*β* = −0.03, 95% CI = −0.06, 0.00; *β* = −0.03, 95% CI = −0.11, 0.04, *p* = 0. 737; *β* = −0.19, 95% CI = −0.32, −0.05; *β* = −0.06, 95% CI = −0.10, 0.00, *p* = 0. 066) (see Figure 4). There was a statistically significant publication bias in the funnel plot (see Supplementary Figure 3) and Egger’s test (*p* = 0.02). The pooled effect of replacing SB with SLP was b = −0.05 (95% CI: −0.10 to 0.00; I^2^ = 73.7%, τ^2^ = 0.0041), indicating borderline statistical significance. The 95% prediction interval was wide (−0.25 to 0.15), encompassing both protective and potentially harmful effects. Leave-one-out analyses (see Supplementary Figure 7) revealed fragility, with statistical significance lost upon exclusion of Menegud et al.’s 30-min/day or 60-min/day contrasts. Cook’s distance (see Supplementary Table S11) identified no formally influential studies, though Menegud et al. (60 min/day) showed the highest influence (0.183). Trim-and-fill analysis (see Supplementary Figure 11) imputed one missing study and attenuated the effect to b = −0.04 (95% CI: −0.09 to 0.01), rendering it non-significant. The observed p-curve (see Supplementary Figure 15) indicates moderate evidence against the null hypothesis, with 64% estimated power and a wide confidence interval.

### 3.6 Isotemporal substitution of SLP to MVPA and depression risk

**Figure 5:**
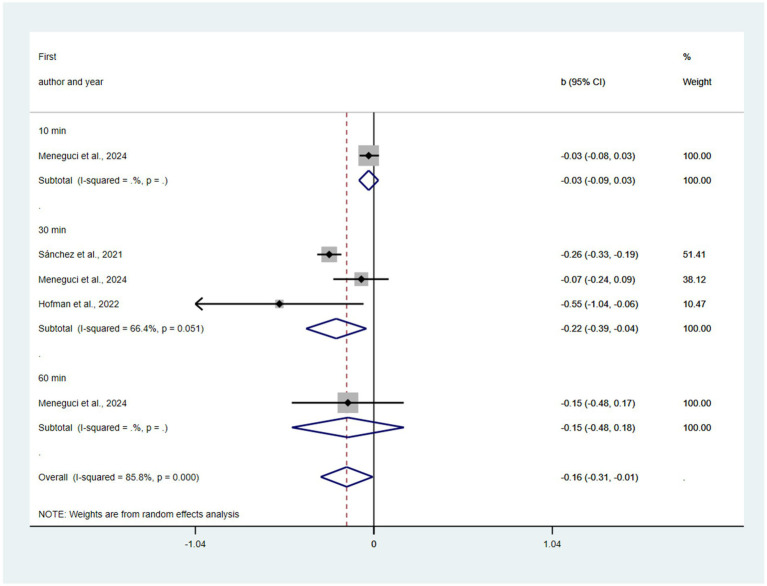
Pooled effect size of substitution of SLP to MVPA on the risk of depression.

Substitution of 10, 30 min, all SLP time with MVPA was predicted to be associated with reductions in depression (*β* = −0.03, 95% CI = −0.09, 0.03; *β* = −0.22, 95% CI = −0.39, −0.04, *p* = 0.051; *β* = −0.15, 95% CI = −0.48, 0.18; *β* = −0.16, 95% CI = −0.31, −0.01, *p* = 0.041) (see Figure 5). There was not a statistically significant publication bias in the funnel plot (see Supplementary Figure 4) and Egger’s test (*p* = 0.29). The pooled effect of replacing SLP with MVPA was b = −0.16 (95% CI: −0.31 to −0.01; I^2^ = 85.8%, τ^2^ = 0.0432), indicating statistical significance. However, the 95% prediction interval was extremely wide (−0.78 to 0.46), encompassing effects ranging from strongly protective to potentially harmful. Leave-one-out analyses (see Supplementary Figure 8) revealed fragility, with statistical significance lost upon exclusion of Cabanas-Sánchez et al. (2021). Cook’s distance (see Supplementary Table S12) identified no formally influential studies (all values < 0.800), though Hofman et al. (2022) showed the highest relative influence (0.148). Trim-and-fill analysis (see Supplementary Figure 12) imputed two missing studies and attenuated the effect to b = −0.08 (95% CI: −0.26 to 0.10), rendering it non-significant. The observed p-curve (see Supplementary Figure 16) shows strong evidence against the null hypothesis, with 99% estimated power and significant right-skewness.

### 3.7 Summary of findings: Isotemporal substitution and depression risk in older adults

Based on the GRADE summary table, substituting 30 min/day SB with MVPA is associated with a statistically significant small reduction in depressive symptoms (*β* = −0.14, 95% CI: −0.25 to −0.02). However, substituting SB with LPA or SLP did not show a significant effect. Similarly, replacing 30 min of SLP with MVPA also yielded a significant beneficial effect (*β* = −0.22, 95% CI: −0.39 to −0.04). The overall certainty of evidence for all findings is LOW-MODERATE (see Supplementary Table S13: GRADE Working Group grades of evidence), primarily due to the observational nature of the included studies (risk of bias), as well as issues of imprecision and inconsistency in specific comparisons (Table 1).

**Table 1:** Summary of findings (SoF) table: substituting SB or SLP with PA for depressive symptoms.

Anticipated absolute effects (95% CI) [Explanation]	Risk with SB	Substitution of 30 min/day of SB with:	№ of participants (studies)	Certainty of the evidence (GRADE)
Depressive symptoms (β coefficient) [The mean depressive symptom score is…]	(Reference)	LPA: −0.00 (−0.08 to 0.08)	18,912 (9)	@@○○ LOW ^a, b^
		MVPA: −0.14 (−0.25 to −0.02)		@@@○ Moderate ^a^
		SLP: −0.03 (−0.11 to 0.04)		@@○○ LOW ^a, c^
Substitution of 30 min/day of SLP with: [The mean depressive symptom score is…]	(Reference)	MVPA: −0.22 (−0.39 to −0.04)	18,912 (9)	@@@○ Moderate ^a^

CI: Confidence Interval; SB: Sedentary Behavior; SLP: Sleep; LPA: Light Physical Activity; MVPA: Moderate-to-Vigorous Physical Activity. ^a^Risk of Bias: Downgraded by one level due to the inherent limitations of the included observational studies (assessed with NOS), which are susceptible to residual confounding. The effect estimates may be influenced by unmeasured variables; ^b^Imprecision: Downgraded by one level for LPA substitution as the 95% CI includes both a negligible effect and a potential small beneficial effect; ^c^Inconsistency: Downgraded by one level for MVPA substitution due to considerable statistical heterogeneity (high I² value) across the studies included in this comparison.

### 3.8 Subgroup analysis

This meta-regression subgroup analysis examined the association between replacing sedentary time with LPA or MVPA and depressive symptoms (see Table 2).

**Table 2:** Results of the subgroup analysis by meta-regression for replacing sedentary time with LPA, MVPA.

Subgroup		Replacing sedentary time with LPA					Replacing sedentary time with MVPA				
		N	β(95% CI)	I^2^ (%)	p^a*^	P^b*^	N	β(95% CI)	I^2^ (%)	p^a*^	P^b*^
All reports		8	0.07(−0.09, 0.23)	97.56	0		10	−0.12(−0.24, –0.01)	91.52	0	
participantscountry	Europe	3	0.25(−0.07, 0.58)	97.54	0	0.2	3	−0.21(−0.55, 0.14)	95.67	0	0.82
	North America	1	−0.04(−0.06, 0.02)	0	0		1	−0.1(−0.14, –0.06)	0	0	
	South America	0	/	/	/		3	−0.13(−0.26, 0)	55.21	0.13	
	East Asia	4	−0.05(−0.15, 0.05)	62.55	0.15		3	−0.01(−0.32, 0.31)	67.32	0.09	
Study design	Cohort	3	0.02(−0.08, 0.12)	0	0.41	0.68	2	−0.31(−0.73, 0.11)	68.53	0.07	0.34
	Cross-sectional	5	0.07(−0.17, 0.32)	99.08	0		8	−0.1(−0.21, 0.02)	90.99	0	
Depression scales	CES-D	1	0.08(−0.34, 0.5)	0	0	0	1	−0.59(−1.06, –0.12)	0	0	0
	GDS	5	−0.02(−0.11, 0.07)	77.01	0		7	−0.14(−0.26, –0.02)	82.02	0	
	PHQ	1	−0.04(−0.06, –0.02)	0	0		1	−0.1(−0.14, –0.06)	0	0	
	Others	1	0.55(0.49, 0.62)	0	0		1	0.06(−0.04, 0.16)	0	0	
PA measures	instrument	7	0.09(−0.09, 0.27)	95.94	0	0.16	6	−0.11(−0.33, 0.12)	91.60	0	0.97
	Self-report	1	−0.04(−0.06, –0.02)	0	0		4	−0.1(−0.17, –0.04)	59.37	0.21	
SB measures	instrument	7	0.09 (−0.09, 0.27)	95.94	0	0.16	6	−0.11 (−0.33, 0.12)	91.6	0	0.97
	Self-report	1	−0.04 (−0.06, –0.02)	0	0		4	−0.1 (−0.17, –0.04)	59.37	0.21	
Sleep measures	Self-report	2	0.07(0.01 0.12)	0	0.94	0.41	2	−0.34(−0.63, –0.04)	50.5	0.16	0.28
	instrument	3	0.15(−0.25, 0.55)	98.74	0		6	−0.07(−0.22, 0.08)	86.68	0.01	
	No report	3	−0.03(−0.17, 0.11)	76.33	0.11		2	−0.1(−0.14, –0.06)	0	0.94	
TIME	10	2	−0.07(−0.13, –0.01)	6.68	0.3	0.08	3	0(−0.28, 0.27)	90.29	0.08	0.29
	30	6	0.12(−0.08, 0.32)	97.96	0		6	−0.14(−0.28, –0.01)	89.81	0	
	60	0	/	/	/	/	1	−0.34(−0.66, –0.03)	0	0	

CES-D, Center for Epidemiologic Studies Depression Scale; GDS, Geriatric Depression Scale; PHQ, Patient Health Questionnaire; P^a^ and P^b^ represent heterogeneity within and between subgroups, respectively.

For LPA replacement, the overall association was non-significant (*β* = 0.07; 95%CI: −0.09, 0.23). Subgroup estimates varied. A positive but non-significant association was observed in European studies (*β* = 0.25), while other regions showed slightly negative estimates. Results also differed by study design and measurement tools, with most confidence intervals crossing zero.

For MVPA replacement, a small, marginally significant negative overall association was found (*β* = −0.12; 95%CI: −0.24, −0.01). This potentially protective effect was consistent across most regions, with the strongest point estimate for Europe (*β* = −0.21). Notably, the effect appeared more pronounced with longer replacement durations (e.g., *β* = −0.34 for 60 min/day). Heterogeneity (I^2^) was high in most subgroups.

In summary, replacing sedentary time with MVPA, but not LPA, showed a weak inverse association with depressive symptoms, with evidence varying across population and methodological subgroups.

## 4 Discussion

Based on our comprehensive review, this appears to be the inaugural meta-analysis to systematically quantify the link between the interchangeability of PA, SB, and SLP with the incidence of depression. Our analysis encompassed 9 observational studies, comprising a cohort of 18,912 individuals (≥ 60 years). The findings suggest that an equivalent reallocation of SB with PA or SLP could be associated with a significant reduction in depression risk. Conversely, the reciprocal substitution of PA or SLP with SB might be correlated with an escalated risk of depression. However, subgroup analysis revealed certain inconsistencies within the data. Additionally, a funnel plot analysis was employed, which provided evidence that some outcomes might be influenced by publication bias. This rigorous approach to data analysis ensures that our conclusions are grounded in a solid evidence base, highlighting the potential impact of lifestyle modifications on mental health outcomes. The observed associations underscore the need for further research to elucidate the mechanisms underlying these relationships and to refine public health strategies aimed at mitigating depression.

The principal outcomes of our meta-analysis indicate that the redistribution of sedentary time in favor of PA and SLP could either decrease or increase the likelihood of developing depression. This suggests that substituting SB with PA and SLP may confer mental health benefits, aligning with the hypothesis that such substitutions could yield more favorable outcomes than more intense forms of activity, which are often harder to integrate into daily routines, particularly in settings like work or education where SB is common (Shrestha et al., 2019). Furthermore, our analysis suggests that LPA and MVPA could serve as viable methods to augment the overall volume of PA, potentially offering additional psychological advantages for those who are already engaged in some form of activity (Kokkinos, 2012). However, the evidence regarding substituting 15 min/day of SB with LPA is inconclusive, as indicated by a non-significant *p*-value. This inconclusiveness may stem from a dearth of studies in this specific time category or from a diminished protective effect of LPA against depression when the substitution period is brief compared to longer durations. Notably, our study detected publication bias affecting the overall effect size estimates for LPA as a replacement for SB. This finding underscores the importance of interpreting these results cautiously, acknowledging the potential for selective reporting and the influence of unpublished data on the observed associations.

Our study utilized isotemporal substitution analysis to estimate the association between the reallocation of time-use behaviors and depression symptoms in older adults during the COVID-19 pandemic. Specifically, we found that substituting SB with LPA and MVPA for periods of 10, 30 min/day, or even the entire duration of SB was significantly associated with reductions in depression symptoms. For instance, increasing MVPA by 15 min/day at the cost of SB was associated with a decrease in depression symptom scores (estimated difference: *β* = −0.13 95%CI −0.17, −0.09). These findings align with previous research indicating that increasing PA and decreasing sedentary time could lower the risk of depression.

Several studies have corroborated the positive impact of replacing SB with PA on reducing depression risk. For example, a study by Su et al. (2022) found that substituting SB with MVPA was associated with a significant decrease in depression symptom scores. Substituting 30 min/day SB with MVPA was associated with a *β* = −0.14 reduction in depression scores (95% CI: −0.25, −0.02). However, it should be noted that this effect magnitude translates to an approximate reduction of only 1.1 points on the PHQ-9 scale, which is below the commonly accepted minimal important difference (MID) of 2–3 points. Similarly, Blodgett et al. (2023) demonstrated that increasing time spent in MVPA relative to other behaviors was associated with a lower risk of depression. Cabanas-Sánchez et al. (2021) also highlighted that replacing 30 min/day of SB with MVPA was beneficially related to depression symptoms. Furthermore, Hofman et al. (2022) observed that substituting SB with MVPA was associated with fewer depressive symptoms. These results collectively underscore the importance of MVPA in reducing the risk of depression and suggest that even relatively short durations of MVPA can have a beneficial effect on mental health.

While the substitution of SB with SLP has shown varying results, there is evidence suggesting that increasing SLP time can mitigate depression symptoms. In our study, substituting SB with SLP was significantly associated with reductions in depression symptoms. This finding is consistent with the results of Meneguci et al. (2024), which indicated that replacing 30 min/day of SB with SLP could reduce depressive symptoms. Similarly, Cabanas-Sánchez et al. (2021) found that replacing SB with SLP was associated with a lower risk of depression. However, Blodgett et al. (2023) noted that increasing SLP at the expense of MVPA was associated with higher depression risk, indicating a complex interplay between different activities and their impact on mental health. Nonetheless, most evidence supports the idea that increasing SLP time could benefit mental health, particularly for those who SLP less than 6 h.

Exploring the impact of substituting PA with SLP on depression symptoms, our findings reveal that reducing MVPA to increase SLP might not always result in beneficial effects on depression. This aligns with the study by Blodgett et al. (2023), which reported that increasing SLP time at the expense of MVPA was associated with a higher risk of depression. Moreover, Cabanas-Sánchez et al. (2021) found that replacing MVPA with SLP was not consistently beneficial, suggesting that maintaining a balance between adequate SLP and sufficient MVPA is crucial. These results highlight the importance of both SLP and MVPA in maintaining good mental health and caution against indiscriminately increasing SLP time at the cost of MVPA.

The practical implications of our findings are substantial. They suggest that interventions aimed at promoting MVPA and reducing SB could effectively mitigate the risk of depressive symptoms among young adults. Theoretically, the results contribute to the growing body of literature emphasizing the importance of considering the entire 24-h activity composition when examining the relationship between movement behaviors and mental health. Understanding these interactions can inform the development of holistic health promotion strategies that simultaneously target multiple aspects of daily activity.

One strength of our study is the use of isotemporal substitution analysis, which allows for examining the effects of substituting one behavior with another within the context of the 24-h day. However, our study has several limitations. Firstly, the cross-sectional design limits our ability to infer causality. Secondly, while we used self-reported measures for time-use behaviors, this method is prone to recall biases. Thirdly, the generalizability of our findings might be limited to the specific population of young adults during the pandemic. Despite these limitations, our study provides valuable insights into the complex relationships between movement behaviors and mental health, paving the way for future research to address these gaps.

A key strength of this review is the application of isotemporal substitution meta-analysis, allowing us to model the theoretical health effects of time reallocation within a finite 24-h day—a more realistic approach than examining behaviors in isolation. However, several limitations must be acknowledged. First, the predominance of cross-sectional studies in the extant literature limits causal inference. Second, measurement heterogeneity (objective vs. self-reported PA, SB, SLP) may introduce bias and complicate pooling. Third, the relatively small number of included studies and high statistical heterogeneity limit the precision and generalizability of some estimates. Fourth, we could not conduct extensive subgroup analyses (e.g., by sex, specific comorbidities) due to data limitations, restricting insights into population subgroups (see Supplementary Figure 17). Finally, while publication bias was assessed, its presence for some outcomes (e.g., LPA substitution) suggests potential influence from unpublished null findings.

Our study has important implications for public mental health. From a public health perspective, these findings underscore MVPA as a priority modifiable behavior for depression prevention in older adults. Interventions should promote replacing sedentary time with MVPA, while also considering the overall 24-h activity composition to avoid detrimental trade-offs (e.g., sacrificing MVPA for excessive SLP). The modest effect size for a 30-min/day MVPA substitution highlights that single behavioral changes may need to be part of a broader, multi-component lifestyle or therapeutic approach for clinically meaningful impact. Future research should prioritize longitudinal and intervention studies to establish causality and determine the dose–response relationship and clinically meaningful thresholds for time reallocation. There is a need for studies using standardized, objective measurement tools and for analyses that explore effect modifiers such as gender, baseline activity level, and clinical status. Finally, investigating the combined and interactive effects of reallocating multiple behaviors within the 24-h cycle will be crucial for developing personalized and effective public health guidelines.

## 5 Conclusion

Our study reveals a significant inverse relationship between MVPA and the risk of depression onset when it replaces SB and SLP, whereas LPA does not exhibit a significant association. Consequently, to bolster the mental health of the elderly, it is advisable to promote interventions that augment moderate-to-vigorous and structured physical activities. Subsequent research should aim to assess the influence of this intervention on the dietary and exercise patterns across diverse sex and age demographics within the older adult population.

## Data Availability

The original contributions presented in the study are included in the article/Supplementary material, further inquiries can be directed to the corresponding authors.
