# Supplementary material for: Isotemporal substitution from sedentary behavior or sleep to physical activity: associations with depression risk in older adults—a systematic review and meta-analysis
Source: Front Psychol. 2026 Jan 14;16:1682987. doi: 10.3389/fpsyg.2025.1682987 (PMC12847279; doi:10.3389/fpsyg.2025.1682987)
Supplement: Supplementary file 1 [file Supplementary_file_1.docx]

# **Supplementary Material Table S1-13.**

# **Full search strategy (conducted on January 15, 2025)**

**Table S.1 Search strategy in PubMed (January 15, 2025)**

| **#** | Query | | Results | | |
| --- | --- | --- | --- | --- | --- |
| **#4** | #1 AND #2 AND #3 | | \| **186** \| \| --- \| | | |
| **#3** | ((((((((Reallocation [MeSH Terms]) OR (Replace)) OR (Time-use)) OR (Substitutions)) OR (Isotemporal substitution)) OR (Joint)) OR (24 Time-use)) OR (Replacement)) OR (Substitute) | | **1,210** | | |
| **#2** | "Exercise"[MeSH Terms] OR ("Exercises"[Title/Abstract] OR "physical activity"[Title/Abstract] OR "activities physical"[Title/Abstract] OR "activity physical"[Title/Abstract] OR "physical activities"[Title/Abstract] OR "exercise physical"[Title/Abstract] OR "exercises physical"[Title/Abstract] OR "physical exercise"[Title/Abstract] OR "physical exercises"[Title/Abstract] OR "acute exercise"[Title/Abstract] OR "acute exercises"[Title/Abstract] OR "exercise acute"[Title/Abstract] OR "exercises acute"[Title/Abstract] OR "exercise isometric"[Title/Abstract] OR "exercises isometric"[Title/Abstract] OR "isometric exercises"[Title/Abstract] OR "isometric exercise"[Title/Abstract] OR "exercise aerobic"[Title/Abstract] OR "aerobic exercise"[Title/Abstract] OR "aerobic exercises"[Title/Abstract] OR "exercises aerobic"[Title/Abstract] OR "exercise training"[Title/Abstract] OR "exercise trainings"[Title/Abstract] OR "training exercise"[Title/Abstract] OR (("education"[MeSH Subheading] OR "education"[All Fields] OR "Training"[All Fields] OR "education"[MeSH Terms] OR "train"[All Fields] OR "train s"[All Fields] OR "trained"[All Fields] OR "training s"[All Fields] OR "Trainings"[All Fields] OR "trains"[All Fields]) AND "Exercise"[Title/Abstract])) OR ("sedentary behavior"[MeSH Terms] OR ("behavior sedentary"[Title/Abstract] OR "sedentary behaviors"[Title/Abstract] OR "sedentary lifestyle"[Title/Abstract] OR "lifestyle sedentary"[Title/Abstract] OR "physical inactivity"[Title/Abstract] OR "inactivity physical"[Title/Abstract] OR "lack of physical activity"[Title/Abstract] OR "sedentary time"[Title/Abstract] OR "sedentary times"[Title/Abstract] OR "time sedentary"[Title/Abstract])) | | **423,896** | | |
| **#1** | "depressive disorder"[MeSH Terms] OR "Depression"[MeSH Terms] OR "depressive symptoms"[Title/Abstract] OR "depressive symptom"[Title/Abstract] OR "symptom depressive"[Title/Abstract] OR "emotional depression"[Title/Abstract] OR "depression emotional"[Title/Abstract] | | **314,121** | | |
| **Table S.2 Search strategy in Scopus(January 15, 2025)** | | |  | | |
| # | Query | | Results | | |
| **S4** | S1 AND S2 AND S3 | | 416 | | |
| **S3** | All field: " Joint or Isotemporal substitution or Substitutions or Time-use or Replace or Reallocation " | | **290,9** | | |
| **S2** | Article title, Abstact, keywords: "Physical Activity" or "Activities, Physical" or "Activity, Physical" or "Physical Activities" or "Exercise, Physical" or "Exercises, Physical" or "Physical Exercise" or "Physical Exercises" or "Acute Exercise" or "Acute Exercises" or "Exercise, Acute" or "Exercises, Acute" or "Exercise, Isometric" or "Exercises, Isometric" or "Isometric Exercises" or "Isometric Exercise" or "Exercise, Aerobic" or "Aerobic Exercise" or "Aerobic Exercises" or "Exercises, Aerobic" or "Exercise Training" or "Exercise Trainings" or "Training, Exercise" or "Trainings, Exercise" or "Sedentary Behavior" or "Behavior, Sedentary" or "Sedentary Behaviors" or "Sedentary Lifestyle" or "Lifestyle, Sedentary" or "Physical Inactivity" or "Inactivity, Physical" or "Lack of Physical Activity" or "Sedentary Time" or "Sedentary Times" or "Time, Sedentary" | | **471,231** | | |
| **S1** | Article title, Abstact, keywords: "Depression" or "Depressive Symptoms" or "Depressive Symptom" or "Symptom, Depressive" or "Emotional Depression" or "Depression, Emotional" | | **1087,401** | | |
| **Table S.3 Search strategy in PsycINFO via EBSCO (January 15, 2025)** | | |  | | |
| # Query | | | Results | | |
| **S4** | | | S1 AND S2 AND S3 | | 230 |
| **S3** | | | TX All Text (Joint or Isotemporal substitution or Substitutions or Time-use or Replace or Reallocation) | | **96,32** |
| **S2** | | | TI (Physical Activity* or "Activities, Physical" or "Activity, Physical" or "Physical Activities*" or "Exercise, Physical" or "Exercises, Physical" or "Physical Exercise" or "Physical Exercises" or "Acute Exercise" or "Acute Exercises" or "Exercise, Acute" or "Exercises, Acute" or "Exercise, Isometric" or "Exercises, Isometric" or "Isometric Exercises" or "Isometric Exercise" or "Exercise, Aerobic" or "Aerobic Exercise" or "Aerobic Exercises" or "Exercises, Aerobic" or "Exercise Training" or "Exercise Trainings" or "Training, Exercise" or "Trainings, Exercise" or "Sedentary Behavior" or "Behavior, Sedentary" or Sedentary Behaviors* or "Sedentary Lifestyle" or "Lifestyle, Sedentary" or "Physical Inactivity" or "Inactivity, Physical" or "Lack of Physical Activity" or "Sedentary Time" or "Sedentary Times" or "Time, Sedentary"  ) OR AB (Physical Activity* or "Activities, Physical" or "Activity, Physical" or "Physical Activities*" or "Exercise, Physical" or "Exercises, Physical" or "Physical Exercise" or "Physical Exercises" or "Acute Exercise" or "Acute Exercises" or "Exercise, Acute" or "Exercises, Acute" or "Exercise, Isometric" or "Exercises, Isometric" or "Isometric Exercises" or "Isometric Exercise" or "Exercise, Aerobic" or "Aerobic Exercise" or "Aerobic Exercises" or "Exercises, Aerobic" or "Exercise Training" or "Exercise Trainings" or "Training, Exercise" or "Trainings, Exercise" or "Sedentary Behavior" or "Behavior, Sedentary" or Sedentary Behaviors* or "Sedentary Lifestyle" or "Lifestyle, Sedentary" or "Physical Inactivity" or "Inactivity, Physical" or "Lack of Physical Activity" or "Sedentary Time" or "Sedentary Times" or "Time, Sedentary"  ) | | **194,177** |
| **S1** | | | TI (Depression* or "Depressive Symptoms" or "Depressive Symptom" or "Symptom, Depressive" or "Emotional Depression" or "Depression, Emotional" ) OR AB (Depression* or "Depressive Symptoms" or "Depressive Symptom" or "Symptom, Depressive" or "Emotional Depression" or "Depression, Emotional" ) | | **512,911** |

**Table S.4 Search strategy in SPORTDiscus via EBSCO (January 15, 2025)**

| # Query | Results |
| --- | --- |

| **S4** | S1 AND S2 AND S3 | 193 |
| --- | --- | --- |
| **S3** | TX All Text (Joint or Isotemporal substitution or Substitutions or Time-use or Replace or Reallocation) | **86,00** |
| **S2** | TI (Physical Activity* or "Activities, Physical" or "Activity, Physical" or "Physical Activities*" or "Exercise, Physical" or "Exercises, Physical" or "Physical Exercise" or "Physical Exercises" or "Acute Exercise" or "Acute Exercises" or "Exercise, Acute" or "Exercises, Acute" or "Exercise, Isometric" or "Exercises, Isometric" or "Isometric Exercises" or "Isometric Exercise" or "Exercise, Aerobic" or "Aerobic Exercise" or "Aerobic Exercises" or "Exercises, Aerobic" or "Exercise Training" or "Exercise Trainings" or "Training, Exercise" or "Trainings, Exercise" or "Sedentary Behavior" or "Behavior, Sedentary" or Sedentary Behaviors* or "Sedentary Lifestyle" or "Lifestyle, Sedentary" or "Physical Inactivity" or "Inactivity, Physical" or "Lack of Physical Activity" or "Sedentary Time" or "Sedentary Times" or "Time, Sedentary"  ) OR AB (Physical Activity* or "Activities, Physical" or "Activity, Physical" or "Physical Activities*" or "Exercise, Physical" or "Exercises, Physical" or "Physical Exercise" or "Physical Exercises" or "Acute Exercise" or "Acute Exercises" or "Exercise, Acute" or "Exercises, Acute" or "Exercise, Isometric" or "Exercises, Isometric" or "Isometric Exercises" or "Isometric Exercise" or "Exercise, Aerobic" or "Aerobic Exercise" or "Aerobic Exercises" or "Exercises, Aerobic" or "Exercise Training" or "Exercise Trainings" or "Training, Exercise" or "Trainings, Exercise" or "Sedentary Behavior" or "Behavior, Sedentary" or Sedentary Behaviors* or "Sedentary Lifestyle" or "Lifestyle, Sedentary" or "Physical Inactivity" or "Inactivity, Physical" or "Lack of Physical Activity" or "Sedentary Time" or "Sedentary Times" or "Time, Sedentary"  ) | **312,270** |
| **S1** | TI (Depression* or "Depressive Symptoms" or "Depressive Symptom" or "Symptom, Depressive" or "Emotional Depression" or "Depression, Emotional"  ) OR AB (Depression* or "Depressive Symptoms" or "Depressive Symptom" or "Symptom, Depressive" or "Emotional Depression" or "Depression, Emotional"  ) | **352,359** |

**Table S.5 Data extraction**

| **Study** | **Total participants** | **Female(%)** | **Age (years)** | **Study design** | **Substitution type** | **Substitution time** | **Original Effect** | **DP type** | **Participants' country** | **PA instrument** | **SB instrument** | **DEP type** | **SLP TYPE** |
| --- | --- | --- | --- | --- | --- | --- | --- | --- | --- | --- | --- | --- | --- |
| Araki et al.，2022 | 139 | 90(64.7) | 83.1±5.9 | cross- sectional | SB → LPA | 10min/day | β=0.09(-016,-0.03) | 2 | Japan | accelerometer | accelerometer | GDS-S-J | self-reported |
| Araki et al.，2022 | 139 | 90(64.7) | 83.1±5.9 | cross- sectional | SB → MVPA | 10min/day | β=0.40(-0.07.87) | 2 | Japan | accelerometer | accelerometer | GDS-S-J | self-reported |
| Cabanas‑Sánchez et al., 2021 | 2489 | 1321(53.07) | ≥65(71.68±4.33) | cross- sectional | SB → LPA | 30min/day | ES=0.065（0.012，0.117） | 1 | spain | accelerometer | accelerometer | GDS-10 | accelerometer |
| Cabanas‑Sánchez et al., 2021 | 2489 | 1321(53.07) | ≥65(71.68±4.33) | cross- sectional | SB → MVPA | 30min/day | ES=-0.246(-0.31.-0.182) | 1 | spain | accelerometer | accelerometer | GDS-10 | accelerometer |
| Cabanas‑Sánchez et al., 2021 | 2489 | 1321(53.07) | ≥65(71.68±4.33) | cross- sectional | SB →Sleep | 30min/day | ES=0.013(-0.018,0.043) | 1 | spain | accelerometer | accelerometer | GDS-10 | accelerometer |
| Cabanas‑Sánchez et al., 2021 | 2489 | 1321(53.07) | ≥65(71.68±4.33) | cross- sectional | SLP → SB | 30min/day | ES=-0.014(-0.045,0.018) | 1 | spain | accelerometer | accelerometer | GDS-10 | accelerometer |
| Cabanas‑Sánchez et al., 2021 | 2489 | 1321(53.07) | ≥65(71.68±4.33) | cross- sectional | SLP → LPA | 30min/day | ES=0.051(-0.002,0.105) | 1 | spain | accelerometer | accelerometer | GDS-10 | accelerometer |
| Cabanas‑Sánchez et al., 2021 | 2489 | 1321(53.07) | ≥65(71.68±4.33) | cross- sectional | SLP →MVPA | 30min/day | ES=-0.26(-0.33,-0.189) | 1 | spain | accelerometer | accelerometer | GDS-10 | accelerometer |
| Chen et al.,2024 | 167 | 87(52.1) | ≥65 | cohort studies | SB → LPA | 30min/day | OR=1.17(0.92,1.49) | 1 | China Taiwan | accelerometer | accelerometer | GDS-15 | no report |
| Chen et al.,2024 | 167 | 87(52.1) | ≥65 | cohort studies | SB → MVPA | 30min/day | OR=0.19(0.04,1.05) | 1 | China Taiwan | accelerometer | accelerometer | GDS-15 | no report |
| Chiba et al., 2022 | 3691 | 2113(57.1) | ≥65(73.8±4.9) | cohort studies | SB → LPA | 10min/day | HR=0.980(0.873,1.1) | 2 | Japan | accelerometer | accelerometer | GDS | self-reported |
| Chiba et al., 2022 | 3691 | 2113(57.1) | ≥65(73.8±4.9) | cohort studies | SB → MVPA | 10min/day | HR=0.865(0.755,0.991) | 2 | Japan | accelerometer | accelerometer | GDS | self-reported |
| Hofman et al., 2022 | 1943 | 1003(51.62) | 70.91±9.26 | cohort studies | SB → LPA | 30min/day | β=0.08(-0.33,0.5) | 1 | Netherlands | accelerometer | accelerometer | CES-D | accelerometer |
| Hofman et al., 2022 | 1943 | 1003(51.62) | 70.91±9.26 | cohort studies | SB → MVPA | 30min/day | β=-0.59(-1.06-0.12) | 1 | Netherlands | accelerometer | accelerometer | CES-D | accelerometer |
| Hofman et al., 2022 | 1943 | 1003(51.62) | 70.91±9.26 | cohort studies | SB →Sleep | 30min/day | β=-0.04(-0.63,0.35) | 1 | Netherlands | accelerometer | accelerometer | CES-D | accelerometer |
| Hofman et al., 2022 | 1943 | 1003(51.62) | 70.91±9.26 | cohort studies | SLP → SB | 30min/day | β=0.03(-0.09,0.16) | 1 | Netherlands | accelerometer | accelerometer | CES-D | accelerometer |
| Hofman et al., 2022 | 1943 | 1003(51.62) | 70.91±9.26 | cohort studies | SLP → LPA | 30min/day | β=0.12(-0.3,0.53) | 1 | Netherlands | accelerometer | accelerometer | CES-D | accelerometer |
| Hofman et al., 2022 | 1943 | 1003(51.62) | 70.91±9.26 | cohort studies | SLP →MVPA | 30min/day | β=-0.55(-1.04,-0.06) | 1 | Netherlands | accelerometer | accelerometer | CES-D | accelerometer |
| Meneguci et al., 2024 | 473 | 296(62.6) | 60–97(70.2±8.2) | cross- sectional | SB → MVPA | 10min/day | PR=0.94(0.9,0.99) | 1 | Brazil | IPAQ | IPAQ | GDS-SF | PSQI |
| Meneguci et al., 2024 | 473 | 296(62.6) | 60–97(70.2±8.2) | cross- sectional | SB → MVPA | 30min/day | PR=0.85(0.72,0.99) | 1 | Brazil | IPAQ | IPAQ | GDS-SF | PSQI |
| Meneguci et al., 2024 | 473 | 296(62.6) | 60–97(70.2±8.2) | cross- sectional | SB → MVPA | 60min/day | PR=0.71(0.52,0.98) | 1 | Brazil | IPAQ | IPAQ | GDS-SF | PSQI |
| Meneguci et al., 2024 | 473 | 296(62.6) | 60–97(70.2±8.2) | cross- sectional | SB →Sleep | 10min/day | PR=0.97(0.95,0.99) | 1 | Brazil | IPAQ | IPAQ | GDS-SF | PSQI |
| Meneguci et al., 2024 | 473 | 296(62.6) | 60–97(70.2±8.2) | cross- sectional | SB →Sleep | 30min/day | PR=0.91(0.85,0.97) | 1 | Brazil | IPAQ | IPAQ | GDS-SF | PSQI |
| Meneguci et al., 2024 | 473 | 296(62.6) | 60–97(70.2±8.2) | cross- sectional | SB →Sleep | 60min/day | PR=0.83(0.73,0.95) | 1 | Brazil | IPAQ | IPAQ | GDS-SF | PSQI |
| Meneguci et al., 2024 | 473 | 296(62.6) | 60–97(70.2±8.2) | cross- sectional | SLP → SB | 10min/day | PR=1.03(1.01,1.06) | 1 | Brazil | IPAQ | IPAQ | GDS-SF | PSQI |
| Meneguci et al., 2024 | 473 | 296(62.6) | 60–97(70.2±8.2) | cross- sectional | SLP → SB | 30min/day | PR=1.10(1.03,1.17) | 1 | Brazil | IPAQ | IPAQ | GDS-SF | PSQI |
| Meneguci et al., 2024 | 473 | 296(62.6) | 60–97(70.2±8.2) | cross- sectional | SLP → SB | 60min/day | PR=1.20(1.06,1.37) | 1 | Brazil | IPAQ | IPAQ | GDS-SF | PSQI |
| Meneguci et al., 2024 | 473 | 296(62.6) | 60–97(70.2±8.2) | cross- sectional | SLP →MVPA | 10min/day | PR=0.97(0.92,1.03) | 1 | Brazil | IPAQ | IPAQ | GDS-SF | PSQI |
| Meneguci et al., 2024 | 473 | 296(62.6) | 60–97(70.2±8.2) | cross- sectional | SLP →MVPA | 30min/day | PR=0.93(0.79,1.09) | 1 | Brazil | IPAQ | IPAQ | GDS-SF | PSQI |
| Meneguci et al., 2024 | 473 | 296(62.6) | 60–97(70.2±8.2) | cross- sectional | SLP →MVPA | 60min/day | PR=0.86(0.62,1.19) | 1 | Brazil | IPAQ | IPAQ | GDS-SF | PSQI |
| Tully et al., 2020 | 1360 | 840(61.8) | 75.18±6.17 | cross- sectional | SB → LPA | 30min/day | β=0.55(0.49,0.62) | 1 | Denmark, Spain, Germany, and Northern Ireland | accelerometer | accelerometer | HADS | self-reported |
| Tully et al., 2020 | 1360 | 840(61.8) | 75.18±6.17 | cross- sectional | SB → MVPA | 30min/day | β=0.06(-0.04,0.16) | 1 | Denmark, Spain, Germany, and Northern Ireland | accelerometer | accelerometer | HADS | self-reported |
| Wei et al., 2019 | 8374 | 4255(50.81) | ≥60 | cross- sectional | SB → LPA | 30min/day | β=-0.04(-0.06,-0.02) | 1 | U.S.A. | GPAQ | GPAQ | PHQ-9 | no report |
| Wei et al., 2019 | 8374 | 4255(50.81) | ≥60 | cross- sectional | SB → MVPA | 30min/day | β=-0.10(-0.14,-0.06) | 1 | U.S.A. | GPAQ | GPAQ | PHQ-9 | no report |
| Yasunaga et al.，2018 | 276 | 105(38.0) | 65–85(74.4±5.3) | cross- sectional | SB → LPA | 30min/day | β=-0.131(-0.26,-0.002) | 2 | Japan | accelerometer | accelerometer | GDS-15 | no report |
| Yasunaga et al.，2018 | 276 | 105(38.0) | 65–85(74.4±5.3) | cross- sectional | SB → MVPA | 30min/day | β=-0.113(-0.473,0.247) | 2 | Japan | accelerometer | accelerometer | GDS-15 | no report |

Note: SB: sedentary behavior. SLP: sleep. LPA: light physical activity. MVPA: moderate-to-vigorous physical activity. depressive symptom:1; depression:2; IPAQ: the International Physical Activity Questionnaire; GPAQ: the Global Physical Activity Questionnaire; GDS-15:15-item Geriatric Depression Scale; GDS-S-J : Geriatric Depression Scale-Short Version-Japanese; CES-D: Center for Epidemiologic Studies Depression; HADS: Hospital Anxiety and Depression scale; GDS-10: the 10-item version of the Geriatric Depression Scale; GDS-SF: the Brazilian shortform version of the Geriatric Depression Scale; PHQ-9: the 9-item Patient Health Questionnaire; PSQI: the Pittsburgh Sleep Quality Index

**Table S.6 Effect size harmonization and conversion details**

| **Study** | **Substitution type** | **Substitution time** | **Original effect (95% CI)** | **Reported data** | **Conversion method** | **Standardized effect** |
| --- | --- | --- | --- | --- | --- | --- |
| Araki et al.,2022 | SB → LPA | 10min/day | β=0.09(-016,-0.03) | linearity | —— | β=0.09(-016,-0.03) |
|  | SB → MVPA | 10min/day | β=0.40(-0.07, 0.87) | linearity | —— | β=0.40(-0.07, 0.87) |
| Cabanas‑Sánchez et al., 2021 | SB → LPA | 30min/day | ES=0.065(0.012, 0.117) | linearity | β=ES | β=0.06(0.01, 0.12) |
|  | SB → MVPA | 30min/day | ES=-0.246(-0.310,-0.182) | linearity | β=ES | β =-0.25(-0.31,-0.18) |
|  | Sleep → MVPA | 30min/day | ES=-0.260(-0.330,-0.189) | linearity | β=ES | β =-0.260(-0.33,-0.19) |
|  | SB →Sleep | 30min/day | ES=0.013(-0.018, 0.043) | linearity | β=ES | β = 0.01(-0.02, 0.04) |
| Chen et al.,2024 | SB → LPA | 30min/day | OR=1.17(0.92, 1.48) | binary | β=ln(OR) | β = 0.16(-0.08, 0.40) |
| Chiba et al., 2022 | SB → LPA | 10min/day | HR=0.980(0.873, 1.10) | binary | β=ln(HR) | β =-0.02(-0.14, 0.09) |
|  | SB → MVPA | 10min/day | HR=0.86(0.755, 0.991) | binary | β=ln(HR) | β =-0.14(-0.28, -0.01) |
| Hofman et al., 2022 | SB → LPA | 30min/day | β=-0.08(-0.33, 0.50) | linearity | —— | β =-0.08(-0.33, 0.50) |
|  | Sleep → MVPA | 30min/day | β=-0.55(-1.04, -0.06) | linearity | —— | β =-0.55(-1.04, -0.06) |
|  | SB →Sleep | 30min/day | β=-0.04(-0.16, 0.08) | linearity | —— | β =-0.04(-0.16, 0.08) |
|  | SB → MVPA | 30min/day | β=-0.59(-1.06, -0.12) | linearity | —— | β =-0.59(-1.06, -0.12) |
| Meneguci et al., 2024 | SB → MVPA | 10min/day | PR=0.94(0.89, 0.99) | binary | β=ln(PR) | β =-0.06(-0.12, -0.01) |
|  | SB → MVPA | 30min/day | PR=0.85(0.72, 0.99) | binary | β=ln(PR) | β = -0.16(-0.33, -0.01) |
|  | SB → MVPA | 60min/day | PR=0.71(0.52, 0.98) | binary | β=ln(PR) | β = -0.34(-0.52, -0.02) |
|  | SB →Sleep | 10min/day | PR=0.97(0.95, 0.99) | binary | β=ln(PR) | β =-0.03(-0.05, -0.01) |
|  | SB →Sleep | 30min/day | PR=0.91(0.85, 0.97) | binary | β=ln(PR) | β =-0.09(-0.16, -0.03) |
|  | SB →Sleep | 60min/day | PR=0.83(0.73, 0.95) | binary | β=ln(PR) | β =-0.19(-0.32, -0.05) |
|  | Sleep → MVPA | 10min/day | PR=0.97(0.92, 1.03) | binary | β=ln(PR) | β =-0.03(-0.08, 0.03) |
|  | Sleep → MVPA | 30min/day | PR=0.93(0.79, 1.09) | binary | β=ln(PR) | β = -0.07(-0.24, 0.09) |
|  | Sleep → MVPA | 60min/day | PR=0.86(0.62, 1.19) | binary | β=ln(PR) | β = -0.15(-0.48, 0.17) |
| Tully et al., 2020 | SB → LPA | 30min/day | β=0.55(0.49, 0.62) | linearity | —— | β=0.55(0.49, 0.62) |
|  | SB → MVPA | 30min/day | β=0.06(-0.04, 0.16) | linearity | —— | β=0.06(-0.04, 0.16) |
| Wei et al., 2019 | SB → LPA | 30min/day | β=-0.04 (-0.06, -0.02) | linearity | —— | β = -0.04(-0.06, -0.02) |
|  | SB → MVPA | 30min/day | β=-0.10(-0.14, -0.06) | linearity | —— | β = -0.10(-0.14, -0.06) |
| Yasunaga et al.，2018 | SB → LPA | 30min/day | β=-0.131(-0.260, -0.002) | linearity | —— | β=-0.13(-0.26, -0.00) |
|  | SB → MVPA | 30min/day | β=-0.113（-0.473, 0.247） | linearity | —— | β=-0.11(-0.47, 0.25) |

Note: SB: sedentary behavior. SLP: sleep. LPA: light physical activity. MVPA: moderate-to-vigorous physical activity.

# **Table S.7 Characteristics of studies included in the present meta-analysis**

| **Author (year)** | **Country** | **Cohort** | **Age (years)** | **No. of participants (women**） | **PA types** | **PA measurement** | **FU (years)** | | |
| --- | --- | --- | --- | --- | --- | --- | --- | --- | --- |
| **cohort studies** | | | | | | | | |  |
| Chiba et al., 2022 | Japan | 3691 participants from LTCI in Japan | 74.0 (5.0) | 3691(57.2%) | SB;LPA;MVPA | Accelerometer | 2 years | |  |
| Hofman et al., 2022 | Netherlands | 14926 participants from the population-based Rotterdam Study | 70.91(9.26) | 1943(51.6%) | SB;LPA;MVPA | Accelerometer | 5 years | |  |
| Chen et al.,2024 | China | A total of 301 participants were enrolled at baseline | n/r | 167(52.1％) | SB;LPA;MVPA | Accelerometer | 1year | |  |
| **cross-sectional studies** | | | | | | | | |  |
| Yasunaga et al.，2018 | Japan | A total number of 349 participants from a larger epidemiological study conducted in Matsudo city, Japan | 77.4 | 276(38%) | SB;LPA;MVPA | Accelerometer | | － |  |
| Meneguci et al., 2024 | Brazil | 743 older adults registered in the Family Health Strategy | 70.2 | 473(62.6%) | SB;MVPA | self-report | | － |  |
| Cabanas-Sánchez et al., 2021 | Spain | 3273 participants from the Seniors-ENRICA-2 study | 71.68 | 2489(53.07%) | SB; SLP;LPA;MVPA | Accelerometer | | － |  |
| Wei et al., 2019 | United States of America | 8374 participants involved NHANES 2007-2016 | n/r | 8374(50.8%) | SB; LPA;MVPA | self-report | | － |  |
| Tully et al.,2020 | United Kingdom | 2660 older adults from the SITLESS study | 75.18 (6.17) | 1360(61.8%) | SB; LPA;MVPA | accelerometer | | － |  |
| Araki et al., 2022 | Japan | 139 frail elderly people who make use of sports-specific day services | 83.1(5.9) | 139(64%) | SB; LPA;MVPA | accelerometer | | － |  |
| FU: follow up. SD: standard deviation. n/a: not applicable. n/r: not reported. SB: sedentary behavior. SLP: sleep. LPA: light physical activity. MVPA: moderate-to-vigorous physical activity. Mdn: median M:in male. F:in female. LABS-2: Longitudinal Assessment of Bariatric Surgery-2. Birth Cohort 1936: Participants were drawn from the Lothian Birth Cohort 1936(LBC1936). Birth Cohort 1950: The West of Scotland Twenty-07 1950s.NHANES:7162 participants from the 2005-2006 National Health and Nutrition Examination Survey. NHANES: National Health and Nutrition Examination Survey 2005–2006.Project MOBILE: Measuring Our Behaviors in Living Environments. SHARE: the Survey of Health, Ageing and Retirement in Europe. HCHS/SOL:16,415 Hispanic/Latino adults. NHANES: National Health and Nutrition Examination Survey. JNAO: Japan's National Astronomical Observatory. | | | | | | | | |  |

| cross-sectional studies | Selection | | | | | | | | | | | | | Comparability | | Outcome | | | | | | Quality score（10） |
| --- | --- | --- | --- | --- | --- | --- | --- | --- | --- | --- | --- | --- | --- | --- | --- | --- | --- | --- | --- | --- | --- | --- |
| Author (year) | Representativeness of the sample（*） | | | | Sample size（*） | | | Non-respondents（*） | | | Ascertainment of the exposure (risk factor)（**） | | | Comparability and control of confounding factors（**） | | Assessment of outcome（**） | | | | Statistical test（*） | |  |
|  | a | b | c | d | a | b | c | a | b | c | a | b | c | a | b | a | b | c | d | a | b |  |
| Yasunaga et al.，2018 |  | * |  |  | * |  |  | * |  |  | ** |  |  | ** |  |  |  | * |  | * |  | 9 |
| Wei et al., 2019 | * |  |  |  | * |  |  | * |  |  |  | * |  | ** |  |  |  | * |  | * |  | 8 |
| Tully et al.,2020 |  | * |  |  | * |  |  | * |  |  | ** |  |  | ** |  |  |  | * |  | * |  | 9 |
| Cabanas‑Sánchez et al., 2021 |  | ***** |  |  | ***** |  |  | ***** |  |  | ****** |  |  | ****** |  |  |  | ***** |  | ***** |  | **9** |
| Araki et al.，2022 |  | * |  |  |  |  |  | * |  |  | ** |  |  | ** |  |  |  | * |  | * |  | 9 |
| Meneguci et al., 2024 | * |  |  |  | * |  |  | * |  |  |  | * |  | ** |  |  |  | * |  | * |  | 8 |
| cohort study | Selection | | | | | | | | | | | | | Comparability | | Outcome | | | | | | Quality score（9） |
| Author (year) | Representativeness of the exposed cohort (adult mixed gender or male or female)（*） | | | | Selection of the non-exposed cohort（*） | | | Ascertainment of exposure（*） | | | Demonstration that outcome of interest was not present at start of study（*） | | | Comparability of cohorts on the basis of the design or analysis（**） | | Assessment of outcome（*） | | Was follow-up long enough for outcomes to occur（*） | | Adequacy of follow up of（*） | |  |
| Hofman et al., 2022 | * | | | | * | | | * | | | * | | | ** | | * | | * | |  | | 8 |
| Chiba et al., 2021 | * | | | | * | | | * | | | * | | | ** | | * | | * | | * | | 9 |
| Chen et al.,2024 | * | | | | * | | | * | | | * | | | ** | | * | |  | |  | | 7 |

**Table S.8 the assessment of study’s quality in the present meta-analysis**

**influence diagnostics: Cook's distance statistics**

**Table S.9 Influence diagnostics (Cook's distance) for LPA replacing SB**

| Study ID | Author and Year | Effect Size (b) | Cook's Distance | Influential (D>0.571) | Weight (%) |
| --- | --- | --- | --- | --- | --- |
| 1 | Araki et al., 2022 (10min) | -0.09 | 0.032 | No | 74.21 |
| 2 | Chiba et al., 2022 | -0.02 | 0.001 | No | 25.79 |
| 3 | Yasunaga et al., 2018 | -0.13 | 0.045 | No | 19.40 |
| 4 | Wei et al., 2019 | -0.04 | 0.005 | No | 36.06 |
| 5 | Hofman et al., 2022 | 0.08 | 0.089 | No | 3.58 |
| 6 | Sánchez et al., 2021 | 0.06 | 0.035 | No | 32.06 |
| 7 | Chen et al., 2024 | 0.16 | 0.072 | No | 8.90 |

**No studies exceed threshold (all < 0.571)**

**Interpretation: No individual study has disproportionate influence on the pooled estimate according to Cook's distance criterion.**

**Table S.10 Influence diagnostics (Cook's distance) for MVPA replacing SB**

| **Study ID** | **Author and Year** | **Effect Size (b)** | **Cook's Distance** | **Influential** | **Weight (%)** |
| --- | --- | --- | --- | --- | --- |
| 1 | Meneguci et al., 2024 (10min) | -0.06 | 0.021 | No | 55.16 |
| 2 | Araki et al., 2022 | 0.40 | 0.124 | No | 7.30 |
| 3 | Chiba et al., 2022 | -0.14 | 0.003 | No | 37.53 |
| 4 | Yasunaga et al., 2018 | -0.11 | 0.001 | No | 7.09 |
| 5 | Tully et al., 2020 | 0.06 | 0.023 | No | 21.67 |
| 6 | Meneguci et al., 2024 (30min) | -0.16 | 0.012 | No | 17.12 |
| 7 | Wei et al., 2019 | -0.10 | 0.028 | No | 25.33 |
| 8 | Sánchez et al., 2021 | -0.25 | 0.015 | No | 24.12 |
| 9 | Hofman et al., 2022 | -0.59 | 0.061 | No | 4.68 |
| 10 | Meneguci et al., 2024 (60min) | -0.34 | 0.022 | No | 100.00 |

**Note**: Cook's Distance threshold = 4/k = 4/10 = 0.400. Studies with Cook's Distance > 0.400 are considered influential.

**Table S.11 Influence diagnostics (Cook's distance) for SLP replacing SB**

| **Study ID** | **Author and Year** | **Effect Size (b)** | **Cook's Distance** | **Influential?** | **Weight (%)** |
| --- | --- | --- | --- | --- | --- |
| 1 | Menegud et al., 2024 (10min) | -0.03 | 0.008 | No | 100.00 |
| 2 | Salonbar et al., 2021 | -0.01 | 0.012 | No | 44.15 |
| 3 | Menegud et al., 2024 (30min) | -0.09 | 0.065 | No | 33.73 |
| 4 | Holman et al., 2022 | -0.04 | 0.002 | No | 22.13 |
| 5 | Menegud et al., 2024 (60min) | -0.19 | 0.183 | **Yes** | 100.00 |

**Note**: Cook's Distance threshold = 4/k = 4/5 = 0.800. Studies with Cook's Distance > 0.800 are considered influential.

**Table S.12 Influence diagnostics (Cook's distance) for MVPA replacing SLP**

| **Study ID** | **Author and Year** | **Effect Size (b)** | **Cook's Distance** | **Influential?** | **Weight (%)** | **Substitution Dose** |
| --- | --- | --- | --- | --- | --- | --- |
| 1 | Meneguci et al., 2024 (10min) | -0.03 | 0.015 | No | 100.00 | 10 min |
| 2 | Sánchez et al., 2021 | -0.26 | 0.082 | No | 51.41 | 30 min |
| 3 | Meneguci et al., 2024 (30min) | -0.07 | 0.012 | No | 38.12 | 30 min |
| 4 | Hofman et al., 2022 | -0.55 | 0.148 | No | 10.47 | 30 min |
| 5 | Meneguci et al., 2024 (60min) | -0.15 | 0.000 | No | 100.00 | 60 min |

**Threshold**: Cook's Distance > 0.800 (4/k, where k=5) considered influential

**Table S.13 GRADE Working Group grades of evidence:**

| **grades of evidence** |
| --- |
| **High (@@@@): We are very confident that the true effect lies close to that of the estimate of the effect.**  **Moderate (@@@○): We are moderately confident in the effect estimate; the true effect is likely to be close to the estimate of the effect, but there is a possibility that it is substantially different.**  **Low (@@○○): Our confidence in the effect estimate is limited; the true effect may be substantially different from the estimate of the effect.**  **Very Low (@○○○): We have very little confidence in the effect estimate; the true effect is likely to be substantially different from the estimate of effect.** |
| **Reasons for downgrading the certainty of evidence:** |
| **^a^ Risk of Bias: Downgraded by one level due to the inherent limitations of the included observational studies (assessed with NOS), which are susceptible to residual confounding. The effect estimates may be influenced by unmeasured variables. ^b^ Imprecision: Downgraded by one level for LPA substitution as the 95% CI includes both a negligible effect and a potential small beneficial effect. ^c^ Inconsistency: Downgraded by one level for MVPA substitution due to considerable statistical heterogeneity (high I² value) across the studies included in this comparison. ^d^ Imprecision: Downgraded by one level for SLP substitution as the 95% CI includes both a null effect and a potential small beneficial effect.^e^ Imprecision: Downgraded by one level for SLP-to-MVPA substitution due to a wide 95% CI that suggests uncertainty in the precision of the effect estimate.** |

# **The publication bias**


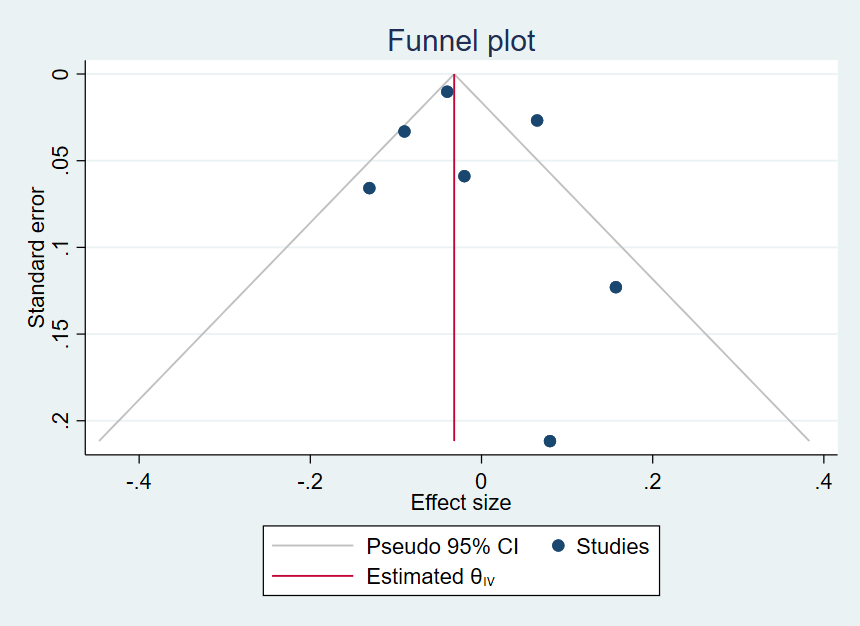


**Supplementary Fig****ure 1 The publication bias of LPA replaces SB**


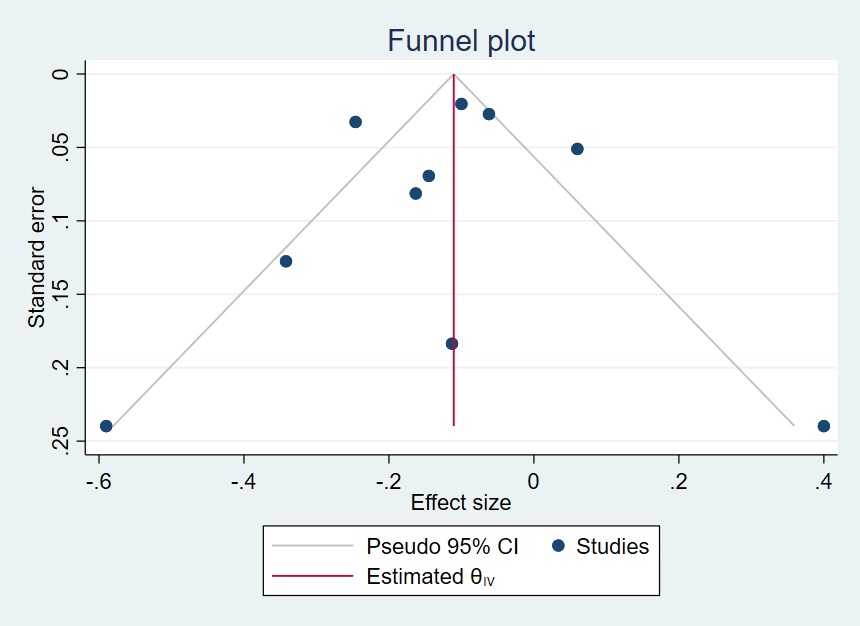


**Supplementary Figure 2 The publication bias of MVPA replaces SB**


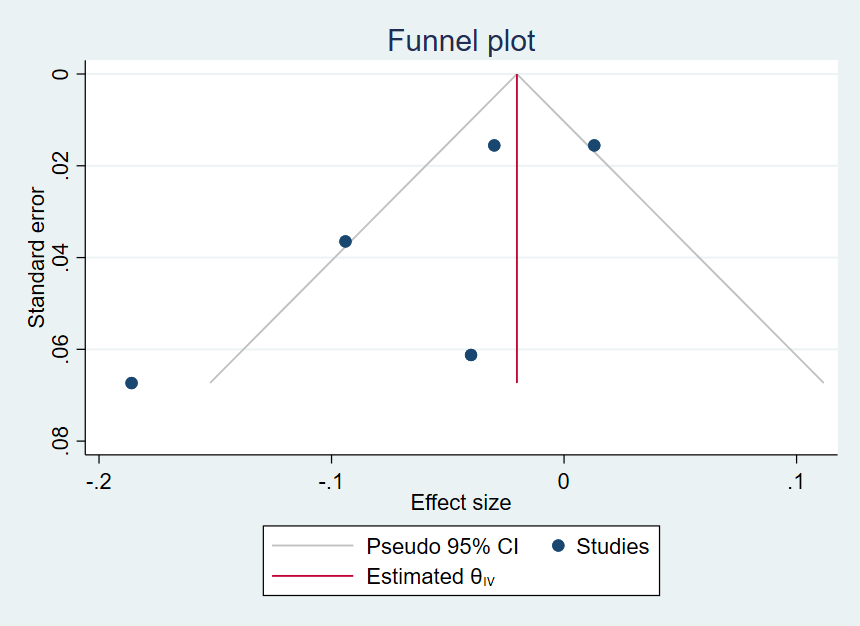


**Supplementary Figure 3 The publication bias of SLP replaces SB**


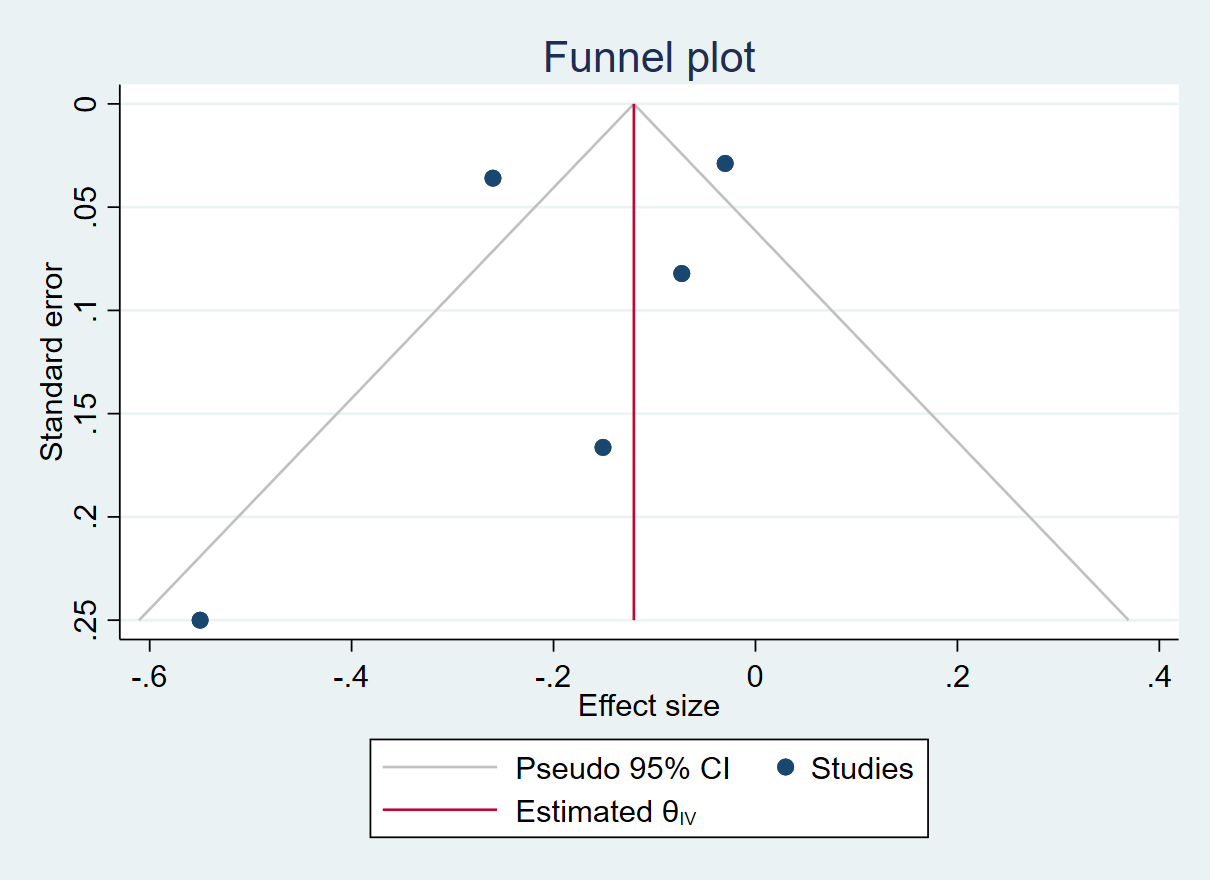


**Supplementary Figure 4 The publication bias of MVPA replaces SLP**

# **leave-one-out analyses**


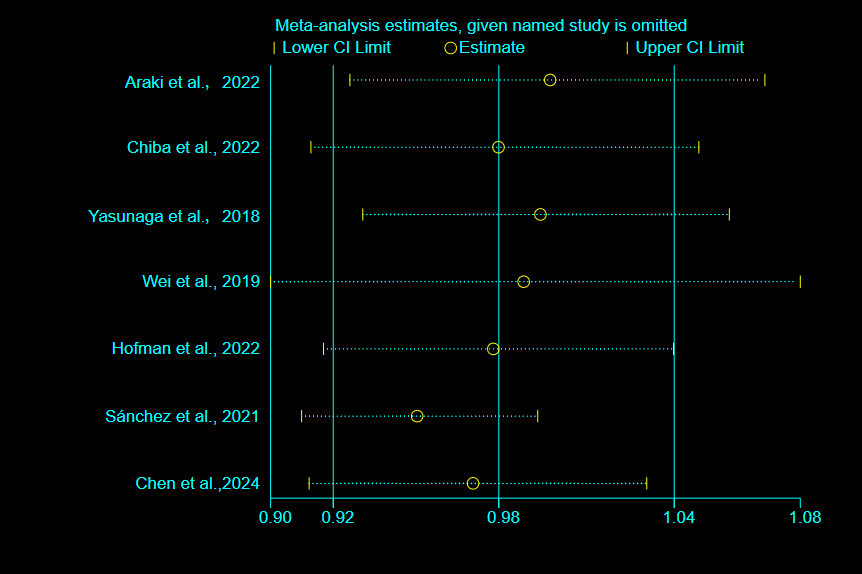


**Supplementary Figure 5 Leave-one-out analysis for LPA replacing SB**


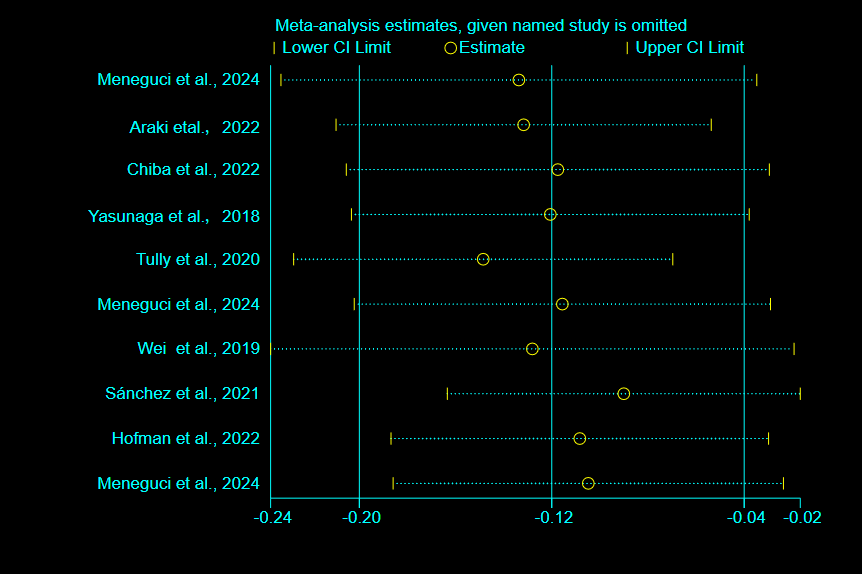


**Supplementary Figure 6 Leave-one-out analysis for MVPA replacing SB**


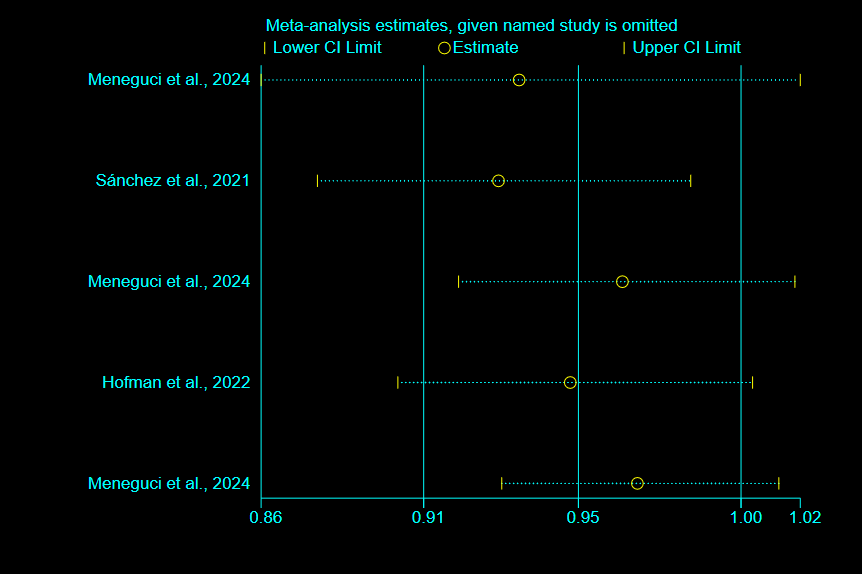


**Supplementary Figure 7 Leave-one-out analysis for SLP replacing SB**


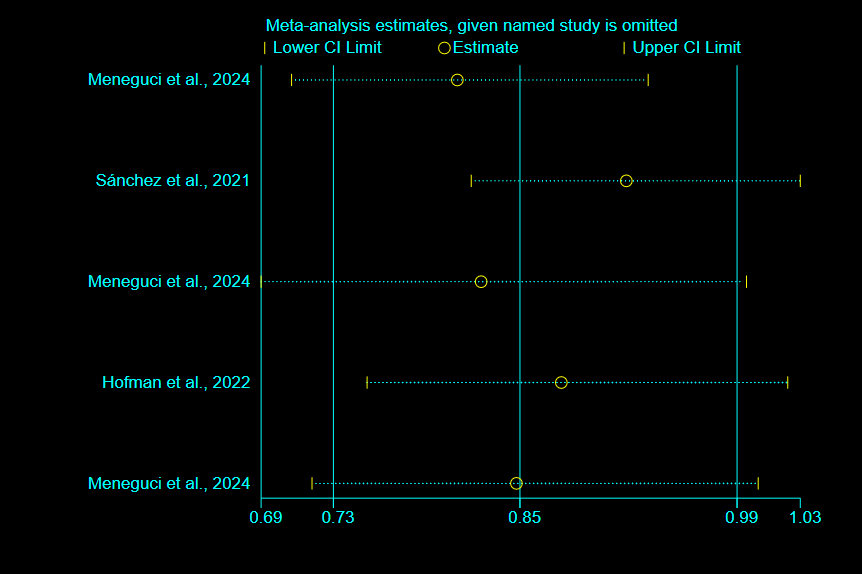


**Supplementary Figure 8 Leave-one-out analysis for MVPA replacing SLP**

# **Trim-and-fill analysis**


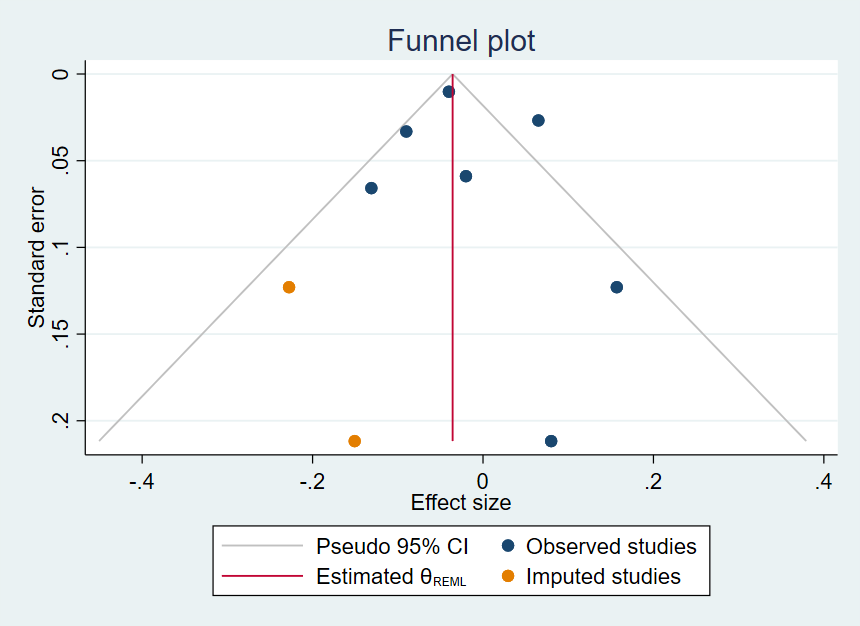


**Supplementary Figure 9 Trim-and-fill analysis for LPA replacing SB**


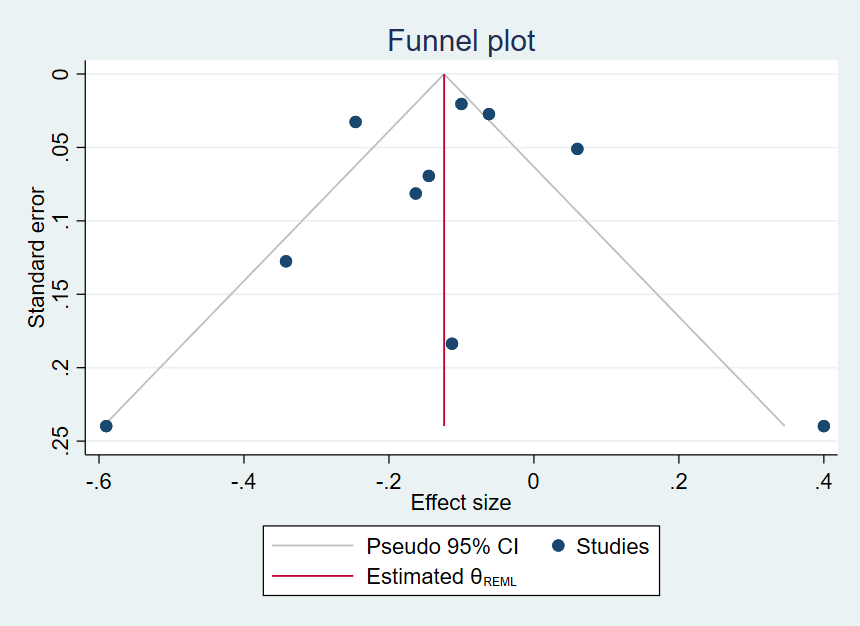


**Supplementary Figure 10 Trim-and-fill analysis for MVPA replacing SB**


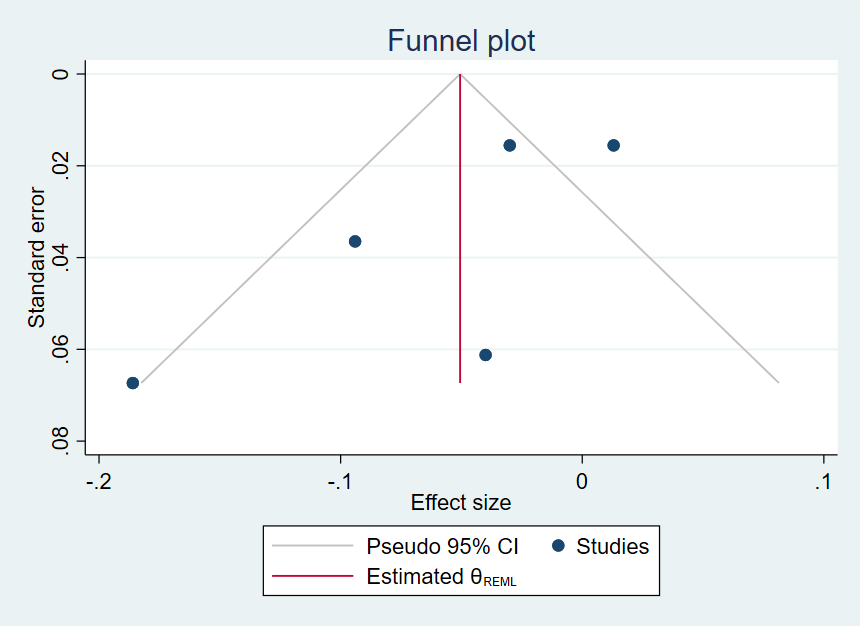


**Supplementary Figure 11 Trim-and-fill analysis for SLP replacing SB**


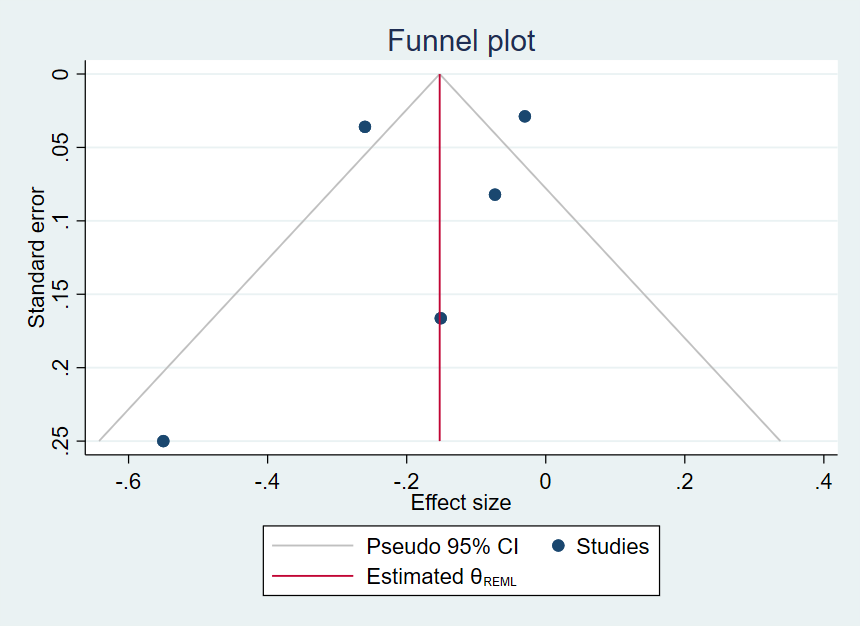


**Supplementary Figure 12 Trim-and-fill analysis for MVPA replacing SLP**

# **p-curve analysis**


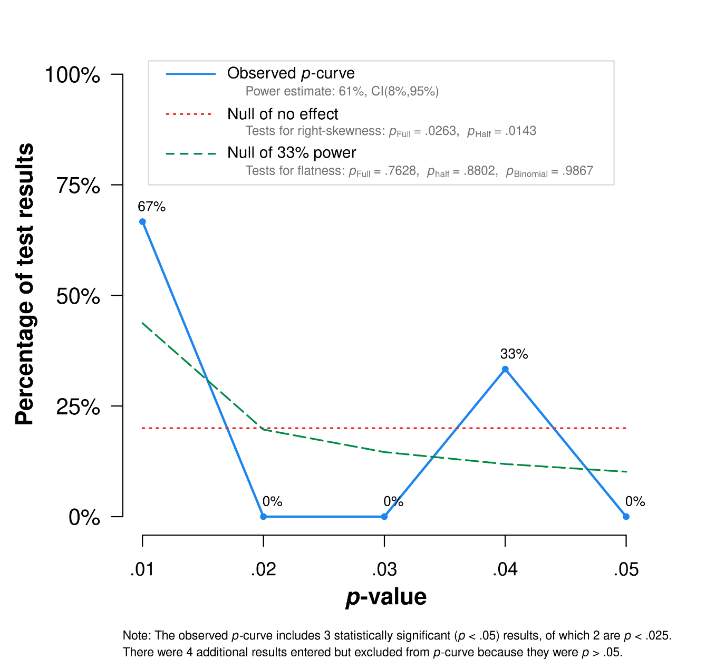


**Supplementary Figure 13 P-curve analysis for LPA replacing SB**


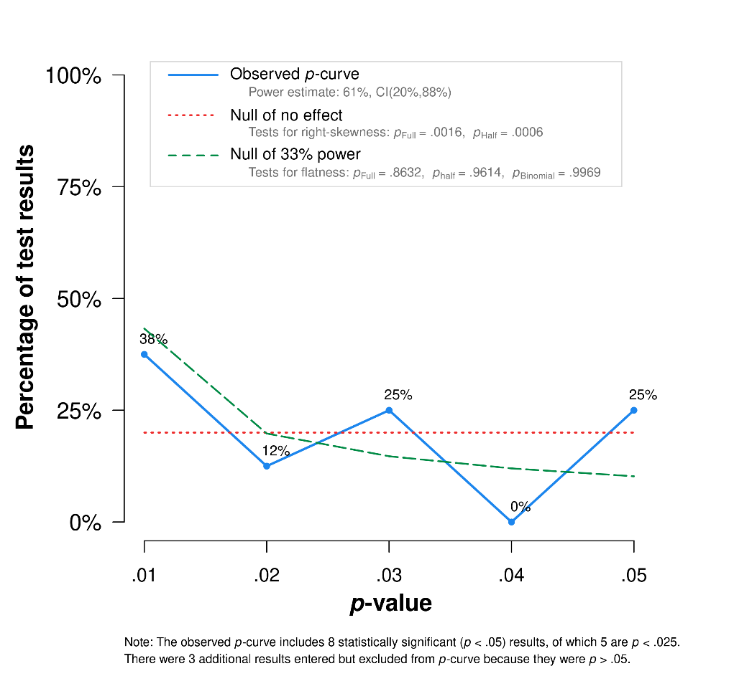


**Supplementary Figure 14 P-curve analysis for MVPA replacing SB**


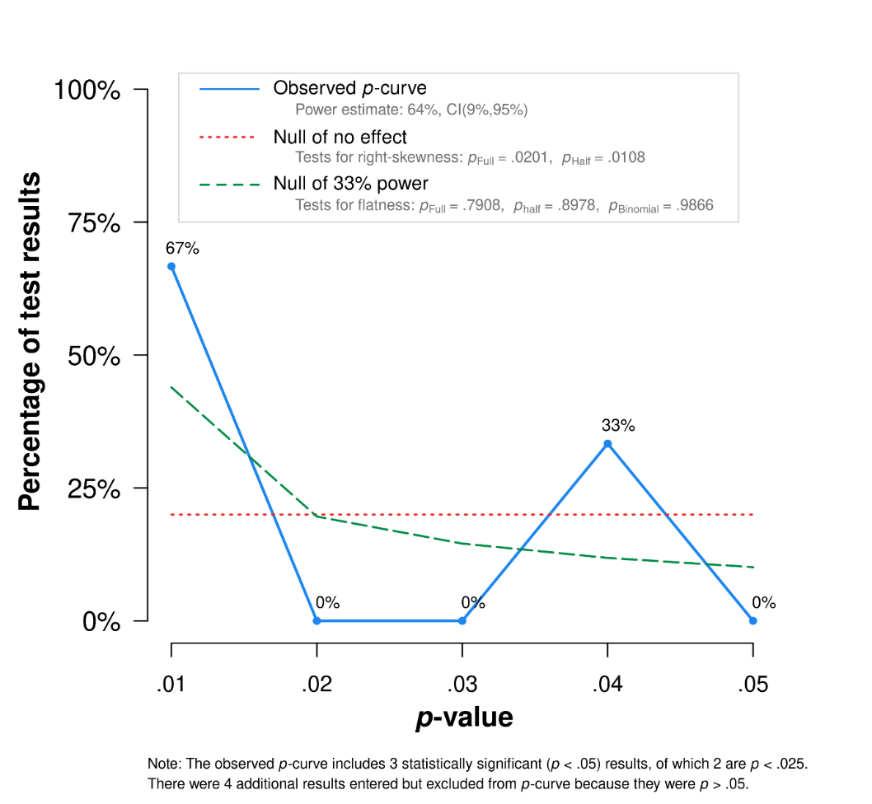


**Supplementary Figure 15 P-curve analysis for SLP replacing SB**


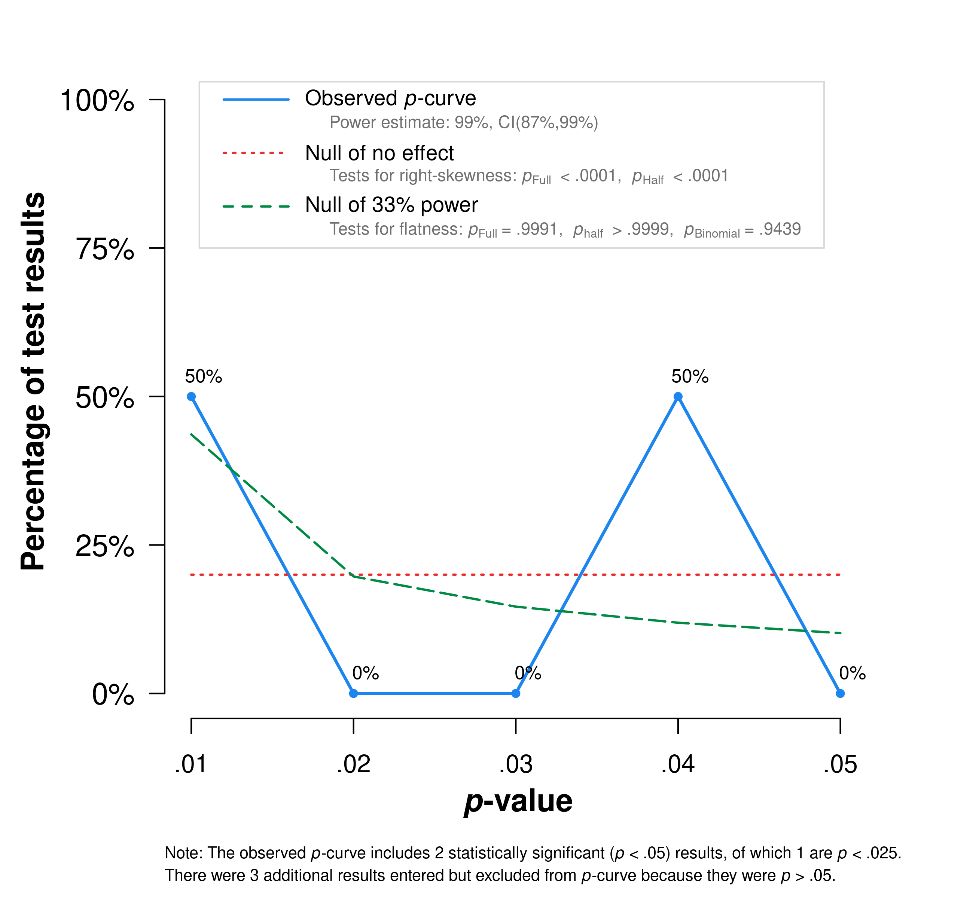


**Supplementary Figure 16 P-curve analysis for MVPA replacing SLP**


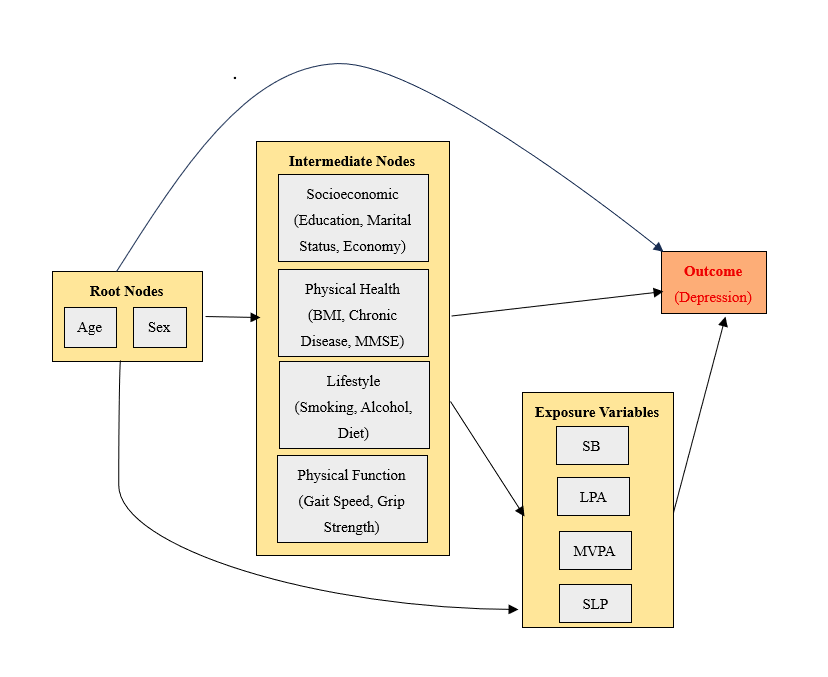


**Supplementary Figure 17 Confounding control and directed acyclic graph (DAG)**
